# Probing Intrinsic Resting-State Networks in the Infant Rat Brain

**DOI:** 10.3389/fnbeh.2016.00192

**Published:** 2016-10-18

**Authors:** Dusica Bajic, Michael M. Craig, David Borsook, Lino Becerra

**Affiliations:** ^1^Center for Pain and the Brain, Boston Children's HospitalBoston, MA, USA; ^2^Department of Anesthesiology, Perioperative and Pain Medicine, Boston Children's HospitalBoston, MA, USA; ^3^Department of Anaesthesia, Harvard Medical SchoolBoston, MA, USA

**Keywords:** blood oxygen level-dependent, BOLD, fMRI, MRI, rs-fMRI, neurodevelopment, neuroimaging

## Abstract

Resting-state functional magnetic resonance imaging (rs-fMRI) measures spontaneous fluctuations in blood oxygenation level-dependent (BOLD) signal in the absence of external stimuli. It has become a powerful tool for mapping large-scale brain networks in humans and animal models. Several rs-fMRI studies have been conducted in anesthetized and awake adult rats, reporting consistent patterns of brain activity at the systems level. However, the evolution to adult patterns of resting-state activity has not yet been evaluated and quantified in the developing rat brain. In this study, we hypothesized that large-scale intrinsic networks would be easily detectable but not fully established as specific patterns of activity in lightly anesthetized 2-week-old rats (*N* = 11). Independent component analysis (ICA) identified 8 networks in 2-week-old-rats. These included Default mode, Sensory (Exteroceptive), Salience (Interoceptive), Basal Ganglia-Thalamic-Hippocampal, Basal Ganglia, Autonomic, Cerebellar, as well as Thalamic-Brainstem networks. Many of these networks consisted of more than one component, possibly indicative of immature, underdeveloped networks at this early time point. Except for the Autonomic network, infant rat networks showed reduced connectivity with subcortical structures in comparison to previously published adult networks. Reported slow fluctuations in the BOLD signal that correspond to functionally relevant resting-state networks in 2-week-old rats can serve as an important tool for future studies of brain development in the settings of different pharmacological applications or disease.

## Introduction

Resting-state functional magnetic resonance imaging (rs-fMRI) provides an important experimental approach to understanding the nature of the brain's intrinsic functional activity in the absence of controlled stimuli or any observed behaviors (Fox et al., 2005; Raichle, 2006). Specifically, rs-fMRI is an imaging procedure that indirectly measures spontaneous neuronal activity by detecting associated changes in blood flow and oxygenation, specifically the blood oxygenation level-dependent (BOLD) signal. Neurovascular coupling (tight coupling between neuronal activity and regional cerebral blood flow) is the physiological basis for the interpretation of the BOLD signal with regard to neural activity (Logothetis et al., 2001; Attwell et al., 2010; Harris et al., 2011; Zehendner et al., 2013). Although rs-fMRI provides only an indirect measure of brain activity mediated by a slow hemodynamic response (Buckner, 1998; Heeger and Ress, 2002; Lindquist et al., 2009), it is now a widely accepted technique for identification of functional connectivity, having high spatial resolution and permitting construction of accurate maps of large-scale functional networks across the brain (Biswal et al., 1995; Hampson et al., 2002; Fox et al., 2005; Thomason et al., 2011). Specific patterns of intrinsic synchronous brain activity are organized as several resting-state networks that are not only present in healthy subjects at different stages of consciousness, but also are reportedly preserved across species (Vincent et al., 2007); see also reviews (Zhang and Raichle, 2010; Rosazza and Minati, 2011; Ganzetti and Mantini, 2013).

However, there is a paucity of information available on resting-state networks in the developing brain across species. Indeed, brain development is a continuous process that takes place not only during gestation, but also long after birth, including during adolescence (Rice and Barone, 2000; Jernigan et al., 2011). Brain plasticity during development is composed of various mechanisms that optimize the integration of sensory stimuli, and are modulated by ongoing synaptic plasticity. Emergence of complex cognitive processes is also supported by maturation and reconfiguration of functional brain networks. Previous studies of human ontogeny of functional connectivity demonstrate that although resting state networks are established as early as the fetal (Thomason et al., 2015), preterm (Doria et al., 2010), and infant period (Fransson et al., 2007; Wylie et al., 2014; Gao et al., 2015), they undergo maturation and refinement over the first two decades of life (Fair et al., 2007, 2008; Gao et al., 2009; Thomason et al., 2011) with age-related increases in within-network functional connectivity (Fair et al., 2007; Thomason et al., 2008; Dosenbach et al., 2010; Uddin et al., 2011; Sherman et al., 2014). Resting-state networks may become functional at different developmental time points in different species (see review, Colonnese and Khazipov, 2012). For rodent development, it is reported that the proliferation and migration of cells in rats begin at gestational day 9.5 and end at about postnatal day (PD)15 (Rice and Barone, 2000). Data from infant rat models demonstrated that in the absence of any stimulation, significant neuronal activity of somatosensory (Colonnese and Khazipov, 2012), as well as prefrontal (Brockmann et al., 2011) cortex matures during the first 1–2-weeks of life. The latter study also indicated that tight network maturation of prefrontal cortex with subcortical structures (e.g., hippocampus) is correlated to transient coupling in oscillatory neuronal rhythms. Furthermore, sensory stimulus evokes neural, but not BOLD fMRI signal, in rats younger than PD11, while the earliest significant sensory stimulus evoked BOLD signal was observed at PD13 rat (Colonnese et al., 2008). Such findings suggest that substantial changes in maturation of neural (Colonnese et al., 2008), neurovascular and autoregulatory (McCandlish et al., 1993; Kozberg et al., 2013) systems occur during the first 2-weeks of life in infant rat to establish the neurovascular coupling. Inhibitory and excitatory neurotransmission (Dorrn et al., 2010; Harris et al., 2011) known to have an effect on cerebral vascular perfusion (Peppiatt et al., 2006; Kocharyan et al., 2008; Attwell et al., 2010) is maturing at this early age, as well. Although direct comparison with human brain development cannot be made, there is a consensus that PD1-14 in infant rats roughly extends from the last trimester of pregnancy up to the first few years of postnatal life in humans (Huttenlocher and Dabholkar, 1997; Clancy et al., 2001, 2007).

In this report, we hypothesized that large-scale resting-state networks would be detectable but not fully established or evolved as specific patterns of activity in 2-week-old rats. To test our hypothesis, we implemented independent component analysis (ICA; Beckmann et al., 2005) of synchronous low frequency BOLD rs-fMRI signal recorded in lightly anesthetized infant rats. Given that intrinsic signaling possibly accounts for a large proportion of brain activity (see reviews, Raichle, 2006; Zhang and Raichle, 2010), our findings of resting-state networks in 2-week-old rats provide a basis of the functional architecture of intrinsic activity at this early age. This developing rat model provides an important application for future mechanistic studies of drug effects and/or altered development on large-scale functional networks across the developing rat brain.

## Methods

The Institutional Animal Care and Use Committee at Boston Children's Hospital approved the experimental protocols for the use of vertebrate animals in this study. Experiments were conducted according to the United States Public Health Service Policy on Humane Care and Use of Laboratory Animals, and the guide for the Care and Use of Laboratory Animals (NIH Publications No. 80-23, revised 1996) prepared by the National Academy of Sciences' Institute for Laboratory Animal Research. All efforts were made to minimize the number of animals used and their discomfort. Pregnant rat dams (Sprague Dawley, Sasco; Charles River Laboratories International, Inc., Wilmington, MA, USA) were received on day 18 of gestation and were handled for several days before delivery. Cages with pregnant dams were checked at 9 A.M. and 5 P.M. daily, and pups found at either time were termed 0 days of age. The progeny from 4 litters were used in this study and pups from both sexes were included. Animals were housed with their litters and were maintained on a 12-h light/dark cycle with food and water given *ad libitum*. Animal scanning was done after 2-weeks of life between PD13–17.

### Anesthesia management

**Figure 1:**
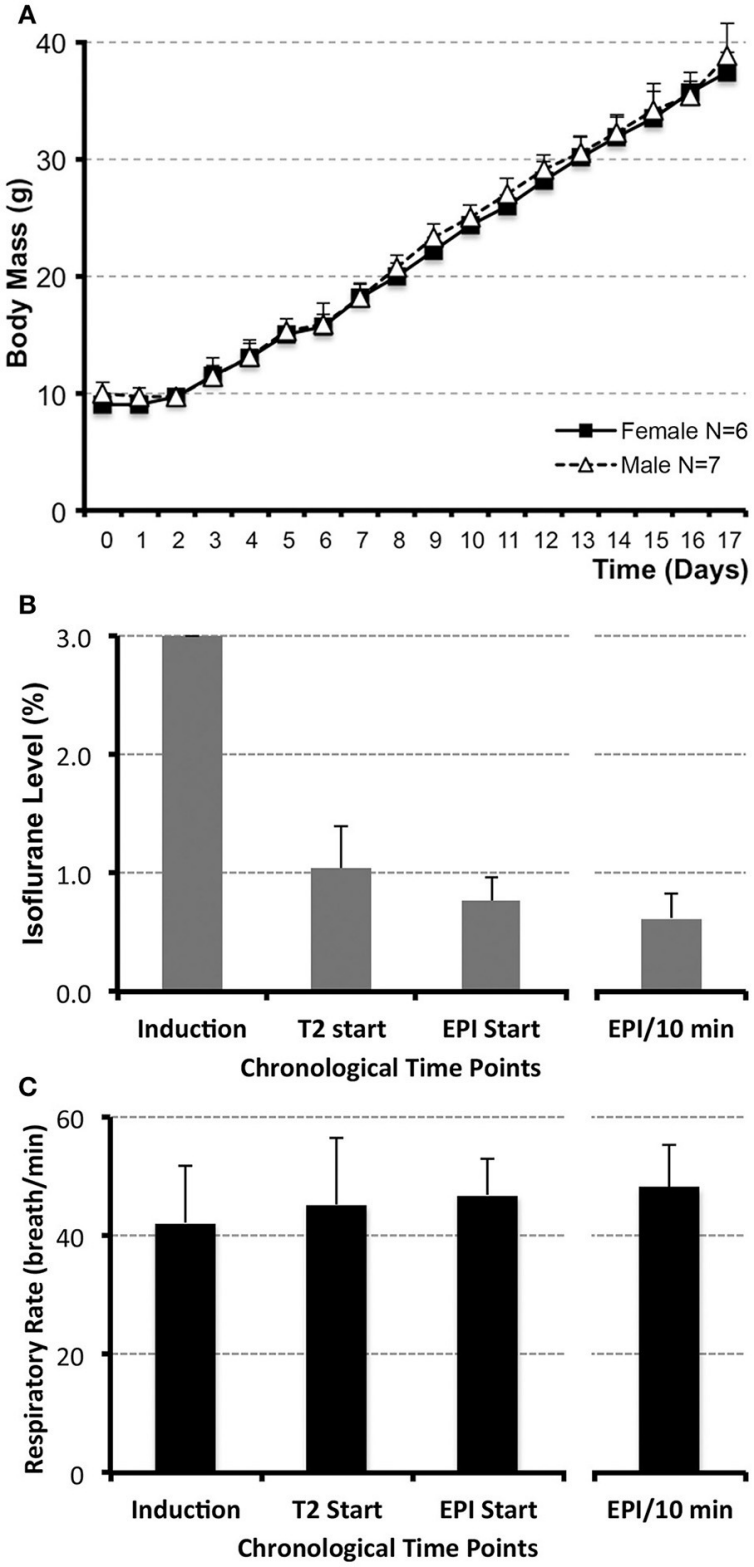
**Infant rat growth (A) and anesthesia management during MRI (B,C)**. **(A)** illustrates the average weight of infant rats (*N* = 13; *g* ± *SD*) from postnatal day (PD) 0, considered the day of birth, through PD17 for both female (*N* = 6) and male (*N* = 7) rats. No significant differences between sexes were observed at any of the time points. **(B)** illustrates average levels of administered inhalational anesthetic, Isoflurane/O_2_ at 1 L/min via nose cone (% ± *SD*; *N* = 13), at different time points during the scanning. Following uniform induction with 3% Isoflurane/O_2_ at 1 L/min, anesthetic level was decreased to maintain a consistent respiratory rate in a narrow range between 45 and 50 breaths/min **(C)**. Analysis included the values at the start of the first (anatomical RARE) scan, as well as the start of the second (functional EPI) scan. Average value of Isoflurane level during the length of the functional scan (based on the two 5 min interval measurements/animal) and its average corresponding respiratory rate are shown in the far-right columns **(B,C)**. Respiratory rate was used as an indirect measure of steady and uniform anesthetic depth during functional scan. No differences in respiratory rate (breath/min ± *SD*) were noted between different time points analyzed [**C**; induction, T2 start, EPI start, average EPI/10 min; *F*_(3, 47)_ = 1.11, *p* = 0.35]. One-way ANOVA with Tukey HSD test.

Rats were anesthetized with Isoflurane (Baxter Healthcare Corp., Deerfield, IL) to minimize stress during scanning and reduce motion-related imaging artifacts. A total of 13 naïve pups (6 female and 7 male from 4 litters) had anesthesia levels monitored during magnetic resonance imaging (MRI). Briefly, infant rats were anesthetized with 3% Isoflurane/O_2_ at 1 L/min up to 3 min prior to being taken to the MRI scanner. Once in the scanner, animals were placed on an animal cradle in a prone position and reconnected to the anesthesia delivery system through a nose cone at the lower anesthesia level, (<1% Isoflurane/O_2_ at 1 L/min). Administration of O_2_ via a nasal cone at 1 L/min provides estimated 24% FiO_2_. No measurement of end-tidal CO_2_ was done being that animals were not intubated. The animal cradle was heated via a long tubing system from a water bath heater (50.4°C) to provide safe, non-specific, mildly warm consistent temperature approved by *Small Animal Imaging Facility* at our institution. The head was secured into a head restrainer with a built-in coil (see below). A respiratory rate monitor was placed on the ventral chest and secured with a paper tape. The whole system was advanced into the magnet. During imaging sessions, the level of anesthesia was gauged by the respiratory rate using the Small Animal Monitoring and Gaiting System (Model 1025-S-50; Instruments Inc., San Diego, CA). The level of Isoflurane was titrated to a respiratory rate in a narrow range between 45 and 50 breaths/min. Following completion of the scan, Isoflurane was discontinued and animals were placed on a warming pad to recover (Hot Dog Patient Warmer; Augustine Biomedical and Design, Eden Prairie, MN). No changes in body color or time to awakening were noted that could implicate alterations in physiological state as a result of changes in internal temperature during scanning. Our goal was to establish (1) anesthesia at the lowest possible level for the functional scan, as well as (2) the steady anesthesia level among all the animals, that will allow for meaningful rs-fMRI group data analysis. To confirm that an identical and steady level of anesthesia was provided among all animals, several endpoints of anesthetic management were quantified. These included mean time values/animal (s ± *SD*) for the anesthesia induction as well as the wake up time. In addition, the average Isoflurane level/animal (% ± *SD*) was evaluated for (1) anesthesia induction, (2) the onset of the anatomical scan, (3) the onset of the functional scan, and (4) the 10 min duration of the functional scan. The same chronological time points were used for evaluation of the average respiratory rate (# breaths/min ± *SD*; Figure 1). Since no differences were found between female and male pups for any of the mean values, data were pulled for statistical analysis. We used a website for statistical computation, Vassar Stats (http://vassarstats.net/), for One-way ANOVA analysis with Tukey HSD test.

### MRI acquisition (imaging)

All scanning was performed with a Bruker BioSpec 70/30USR 7T MRI scanner (Bruker, Billerica, MA) at the Small Animal Imaging Laboratory at Boston Children's Hospital. We used a Bruker inner diameter of 85 mm transmit-only volume coil in combination with an anatomically shaped Bruker mouse brain 4-channel phased array receive-only surface coil (10–20 mm internal diameter; Bruker, Billerica, MA). This is because the size of the infant rats at the 3rd week of life (PD13–17) was equivalent to the size of an adult mouse. This head coil is most appropriate for animals' weight <40 g. A Bruker fastmap shimming program was performed to improve the homogeneity of the B0 field. High-resolution **anatomical** images were acquired with a fast-spin echo sequence (RARE; T2 weighted study) for 4 min 16 s. Excitation pulse was 90 degrees (2.7 ms). We used the following parameters: a field of view of 20 × 20 mm, spatial resolution 0.078 × 0.078 mm, an in-plane resolution of 256 × 256 voxels, slice thickness of 0.5 mm, gap of 0.1 mm, 34 slices, and RARE factor 8. Images were acquired with repetition time (TR) = 4000 ms and echo time (TE) = 35 ms. Subsequently, a 10-min functional scan was obtained with co-centered single-shot BOLD rs-fMRI time series using an echo planar imaging (EPI) sequence. Used EPI sequence was a gradient echo (GRE). The following EPI parameters were used: a field of view of 20 × 20 mm, spatial resolution of 0.313 × 0.313 mm, an in-plane resolution of 64 × 64 voxels, slice thickness of 0.75 mm, gap of 0.15 mm, and 20 slices. EPI images were acquired with *TR* = 1000 ms, *TE* = 37.323 ms, and 600 volumes per animal.

### Resting-state networks analysis

Preprocessing and statistical analyses were performed using tools from the FMRIB software library (http://www.fmrib.ox.ac.uk/fsl/) and custom scripts written in MATLAB as previously described (Becerra et al., 2011) and detailed below.

#### Preprocessing

**Figure 2:**
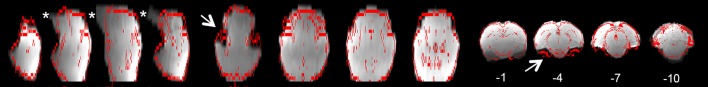
**Representative registration of 2-week-old rat using an adult rat template**. Figure illustrates representative individual functional-to-standard registration of all 2-week-old rats included in the ICA analysis. The gray image represents individual rs-fMRI data while the red contour represents the outline of an adult atlas as reported by FSL output. First four columns are in axial view; the next four in sagittal, and the remaining four columns represent coronal view. Common expected artifact is seen in the ventral regions of the rat brain (near ear channels; Schwarz et al., 2006). It is best visualized in the first transverse and the second coronal section (arrows). Distortions noted in ventral parts of the brain were noted in all individual rats only in the caudal region of the brainstem (stars). Numbers below coronal slices represent distance from Bregma (mm). Section with Bregma of 0 mm corresponds to Panel 17 of Rat Brain Atlas (Paxinos and Watson, 1998). Left hemisphere of the brain corresponds to the right side of the image.

Several preprocessing steps were carried out prior to brain network analysis. All images had their pixel dimensions scaled up in the Nifti header by a factor of 10 to avoid scale-depended issues when using standard FSL software. With the exception of brain extraction and band-pass filtering, all steps were carried out in the MELODIC graphic user interface. Preprocessing steps included: (i) *Brain extraction*: Brain extraction was done manually. Specifically, masks were created manually by masking all 20 slices from the first volume of each individual rat to generate a mask file. This mask file was then applied to the 600 volumes in each functional image. (ii) *Band-pass filtering*: Functional images were band-pass filtered between 0.01 and 0.1 Hz. (iii) *Slice timing correction*: Interleaved slice timing correction was used because each slice was acquired in interleaved order (0, 2, 4 …1, 3, 5 …). (iv) *Spatial smoothing*: Functional data was spatially smoothed to smooth out minor registration imperfections. Because we are interested in large-scale networks across the whole brain of a young rat, we applied a Gaussian kernel FWHM of 0.7 mm to the preprocessed data to identify relatively large areas of coherent activity. (v) *Normalization to standard space*: Being that animals slightly differ in brain size, individual brains were registered to a standardized anatomical image after running brain network analysis (see below). Registration of rs-fMRI data to a standard space (in-house adult anatomical rat brain template) was carried out using FSL's *flirt* using 12° of freedom affine transformation and a resampling resolution of 0.4 mm. In other words, affine transformation for registration was used to inspect for the proper alignment of each individual rat to the adult rat brain atlas. This step is a pre-requisite to performing group analysis and identifying common networks across all animals. Common expected minimal artifact was seen across all the animals in the ventral regions of the brain (near ear canals; Schwarz et al., 2006). In addition, registration was consistently and uniformly distorted in the ventral parts of caudal brainstem across all brains (Figure 2). As a result, data from medulla regions (distal to Bregma value of −11 mm) was not displayed nor included in results.

#### MRI data quality assessment

**Figure 3:**
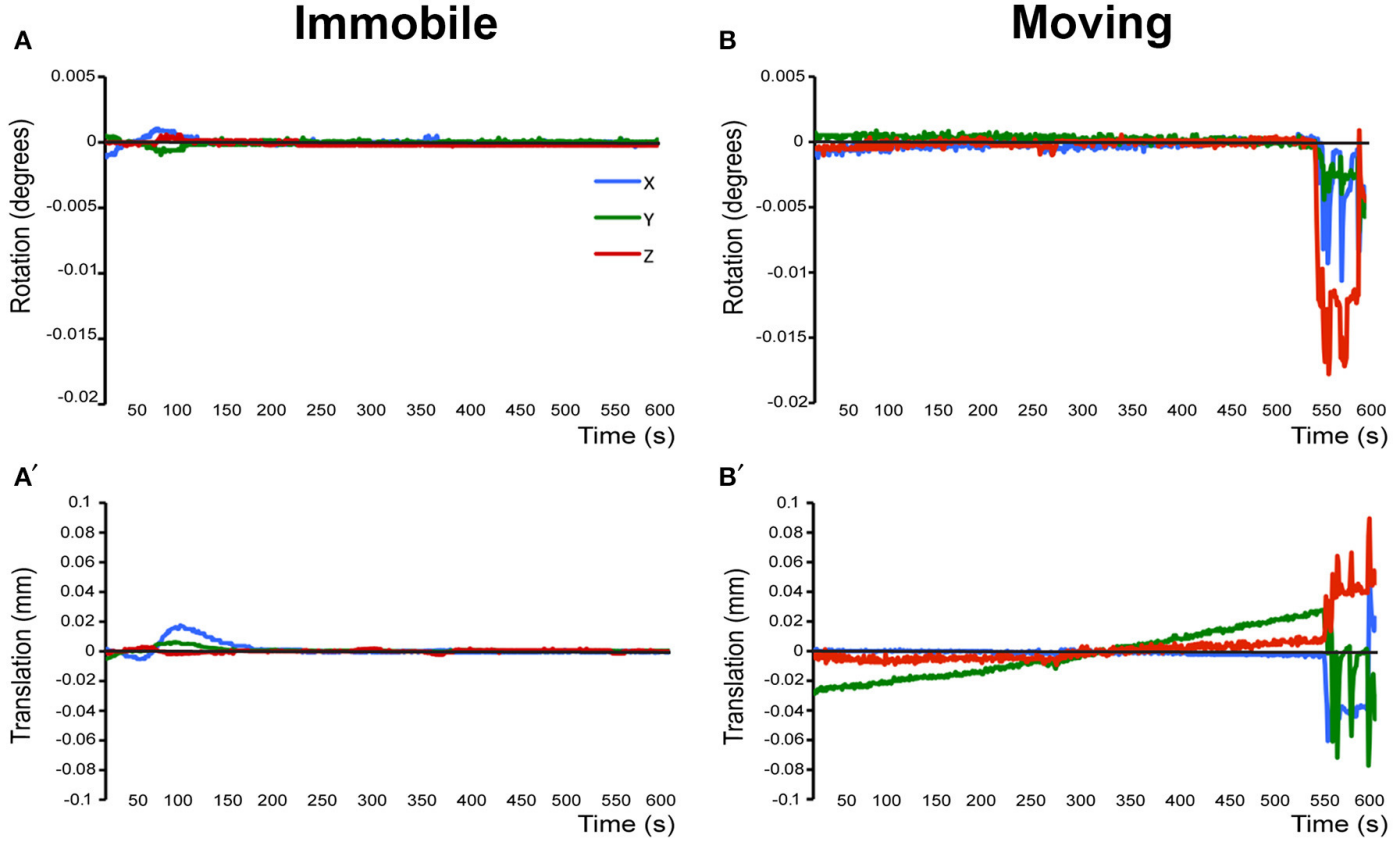
**Assessment of motion in lightly anesthetized infant rats during imaging. (A,A')** display the rotation (in degrees) and translation (in mm) for an immobile 2-week-old rat during MRI, respectively. Rotation is a rigid body movement and refers to the movement of the head around a center point. Translation is every point on the head moving a constant distance in a specific direction. The immobile rat's head did not rotate more than 0.005 degrees or moved more than 0.02 mm. This is an acceptable amount of movement for the group ICA. **(B,B')** illustrate rotation and translation of an infant rat that moved during the scanning, which lead to a motion-related imaging artifact. As a result, data obtained from this animal was excluded from the group ICA. Blue X line, horizontal axis; Green Y line, vertical axis; Red Z line, longitudinal axis of the scanner.

Prior to running the final group preprocessing steps and brain network analysis, all rats were assessed for full brain coverage and motion artifacts by running a single subject independent component analysis (ICA; see below). If a scan showed only partial brain coverage or excessive motion artifacts (>½ voxel size), the animal was excluded from the subsequent group analysis. Specifically, only 1 out of 13 animals was excluded from final analysis because of partial brain coverage during scanning due to misplacement of the receiver coil (not shown). A second rat was excluded from the final analysis due to motion-related imaging artifacts as illustrated in Figure 3. The remaining 11 animals did not show excessive motion and, therefore, were included in the final group ICA analysis.

#### Brain network analysis

In this report, resting-state brain network data analysis was performed using spatial ICA (Stone, 2002; Beckmann et al., 2005; Cole et al., 2010) with the MELODIC tool from FSL. ICA is a data-driven statistical method that analyzes the entire set of rs-fMRI signals to derive a set of components (networks) that are maximally independent in the spatial domain. As previously described (Svensén et al., 2002), group ICA estimates a common set of components or networks for the whole group so that possible subtle differences between subjects (e.g., sex difference) may be lost. It included several steps of the analysis: (i) *Variance normalization*: Timecourses were variance-normalized to rescale each time series so that estimation is primarily influenced by the voxel-wise temporal dynamics and not by a voxel's amplitude signal. (ii) *Spatial ICA*: We used multi-session temporal concatenation to implement our ICA. This type of ICA allows for the processing of several subjects simultaneously while constraining the spatial maps to be identical across subjects with no constraints on the timecourses for each subject. Our analysis was set to extract 40 components from the rs-fMRI data. Forty components seem to be a reasonable number to provide sub-network separation without incurring into mathematical granularity that produces individual structures as components (Hutchison et al., 2010; Liang et al., 2011). In performing temporal concatenation across several rats, we provided MELODIC with a standards anatomical brain used in our previous adult rat imaging studies (Becerra et al., 2011). (iii) *Comparison to standard adult rat brain networks*: All 40 extracted components were then spatially correlated with Pearson's *r* to seven previously identified adult rat brain template networks (Becerra et al., 2011). This step aided in the identification of networks that showed high spatial similarity to adult template networks (Table 1). For further analysis, we also included other networks that did not correlate with the adult templates (Pearson's correlation of *r* < 0.2) but had spatial distribution that reflected network activity. These networks were closely inspected to eliminate networks associated with artifacts or physiological noise. (iv) *Dual Regression*: The set of spatial maps from the group average analysis was used to generate subject-specific versions of the spatial maps and associated timeseries, using dual regression (Filippini et al., 2009). Finally, we tested for group average using FSL's *randomize* permutation-testing tool. (v) *Registering and Masking network images*: After components were identified, they were transformed through a unitary transformation matrix back to standard (resolution) space. In other words, before proceeding to the next step we registered each network image from low resolution standard space to high resolution standard space and mask images in order to eliminate non-brain activity. (vi) *Thresholding statistical spatial maps*: These maps were thresholded using an alternative hypothesis test based on fitting a Gaussian/gamma mixture model to the distribution of voxel intensities within spatial maps (Beckmann et al., 2005). We used a mixture model analysis approach for inference (Pendse et al., 2009). The lowest threshold is marked in the heat map for each network. (vii) *Identifying brain clusters comprising individual networks*: This step determines the size and location of anatomical areas (clusters) comprising individual networks. Brain regions were defined using a standard rat brain atlas (Paxinos and Watson, 1998). We used an in-house MATLAB program to define clusters. The program uses a “watershed” approach to identify and separate clusters of activity. The program reports the peak statistical value of the cluster with its brain coordinates and its volume as provided in Supplemental Table 1. Network identification and terminology was guided by correlating each component's spatial layout with seven previously established adult resting-state brain networks (Becerra et al., 2011).

**Table 1:** **Components comprising resting-state networks of 2-week old rat in relation to previously described adult rat networks**.

**Adult template network**	**Independent component number**	**Pearson's *r* correlation**
Default mode network	2	0.20
	7	0.34
Sensory (Exteroceptive)	4	0.33
	10	0.27
	12	0.27
Salience (Interoceptive)	1	0.35
	3	0.24
^*^Basal ganglia—hippocampus	22	0.20
	31	0.36
Autonomic	25	0.27

*To identify networks that showed high spatial similarity to adult networks, all 40 extracted components in 2-week old rats were spatially correlated with Pearson's r to seven previously identified adult rat brain template networks (Becerra et al., 2011). Table lists correlation to five described resting-state networks in adult rats. With the exception of Autonomic network, there are several components for each network in 2-week old rat. Pearson's r correlation (≥0.2) is listed for each individual component. For detailed explanation, see Methods Section*.

* *We describe Basal Ganglia network in the 2-week old rat that we correlate to Basal Ganglia-Hypothalamus network of the adult rat*.

## Results

### Anesthesia level

There was no difference in body mass between female (*N* = 6) and male (*N* = 7) infant rats during the first 2-weeks of life (PD1–17; Figure 1A). Our goal for anesthetic management was to establish the steady level of anesthesia at the lowest % of Isoflurane during the duration of functional scan. Initially, anesthesia induction was performed with 3% Isoflurane/O_2_ at 1 L/min for 2.73 min ± 0.44 (*N* = 13). Subsequently, anesthetic was decreased so that average Isoflurane level at the onset of the first (anatomical RARE) scan was 1.04% ± 0.35, and at the onset of the second (functional EPI) scan was 0.76% ± 0.21. Average value of Isoflurane level during the length of the functional scan (based on the two 5 min interval measurements/animal) was 0.62% ± 0.21 (Figure 1B). Respiratory rate (breaths/min ± *SD*) was used to gauge the Isoflurane titration and served as an indirect measure of the anesthetic depth. Respiratory rate stayed consistent throughout the scan (Figure 1C). No differences in respiratory rate were noted between different time points analyzed [*F*_(3, 47)_ = 1.11, *p* = 0.35]: induction (43 breaths/min ± 10), T2 start (45 breaths/min ± 11), EPI start (47 breaths/min ± 6), average EPI/10 min (48 breaths/min ± 7). Such anesthesia management provided successful immobility during scanning session; only 1 out of 13 animals was excluded from the subsequent rs-fMRI analysis due to motion-related imaging artifacts; see below. Following completion of scanning and termination of Isoflurane administration, animals woke up within the 1st min (0.15 min ± 0.32). Presented anesthesia management data implies that animals were sedated uniformly and adequately with steady low levels of Isoflurane (<1%) among all animals for the duration of the functional scan.

### Resting-state networks identification in infant rats

**Figure 4:**
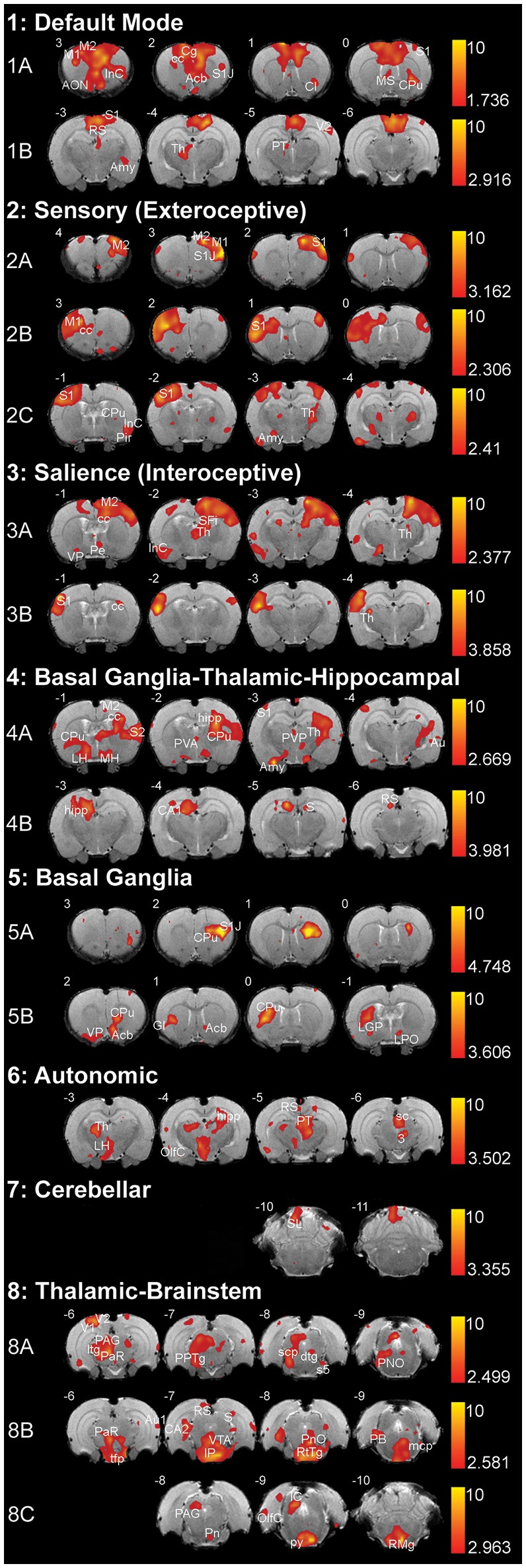
**Resting-state networks in 2-week-old rats**. The figure illustrates eight different resting-state networks that were identified in 2-week-old rats (*N* = 11; 5 female and 6 male from 4 different litters) under light anesthesia. Networks 1–7 showed some spatial similarity to adult networks and included: **(1)** Default Mode Network, **(2)** Sensory (Exteroceptive) Network, and **(3)** Salience (Interoceptive) Network, **(4)** Basal Ganglia-Thalamic-Hippocampal Network, **(5)** Basal Ganglia, **(6)** Autonomic, and **(7)** Cerebellar Network. The last, **(8)** Thalamic-Brainstem Network has not been described in adult rats. With the exception of Autonomic and Cerebellar Networks, all others were comprised of multiple components extracted by Melodic. The resting-state networks maps are represented as *z*-scores. The threshold bars show a maximum threshold of 10 and the minimum *z*-score necessary for significance for each component. The positive map colors range from dark red to yellow. The numbers in the upper left corner of each coronal section refer to distance from Bregma (mm). Each individual coronal section corresponds to traditional radiographic orientation; the left hemisphere of the brain corresponds to the right side of the image. Anatomical abbreviations were adopted from Rat Brain Atlas (Paxinos and Watson, 1998). 3, oculomotor nucleus; Acb, accumbens nucleus; Amy, amygdala; AON, anterior olfactory nucleus; Au1, primary auditory cortex; Au, auditory cortex; CA1; field CA1 of hippocampus; CA2; field CA2 of hippocampus; Cb6, cerebellar lobule 06; Cb7, cerebellar lobule 07; Cb8, cerebellar lobule 08; cc; corpus callosum; Cg, cingulate cortex; Cl, claustrum; CPu, caudate putamen; dtg, dorsal tegmental bundle; GI, granular insular cortex; hipp, hippocampus; ic, internal capsule; IC, inferior colliculus; IP, interpeduncular nucleus; InC, insular cortex; LGP, lateral globus pallidus; LH, lateral hypothalamus; LPO, lateral preoptic area; M1, primary motor cortex; M2, secondary motor cortex; mcp, medial cerebellar peduncle; MH, medial hypothalamus; MS, medial septal nuclei; OlfC, olfactory cortex; PAG, periaqueductal gray; PaR, pararubral nucleus; PB, parabrachial nucleus; Pe, periventricular hypothalamic nucleus; Pir, piriform cortex; PnO, pontine reticular nucleus, oral part; PPTg, pedunculopontine tegmental nucleus; PT, pretectum; PVA, paraventricular thalamic nucleus—anterior; PVP, paraventricular thalamic nucleus; py, pyramidal tract; RS, retrospenial cortex; RtTg, reticular tegmentum; S, subiculum; S1, primary somatosensory cortex; S1J, primary somatosensory cortex—jaw region; S2, secondary somatosensory cortex; s5, sensory root of trigeminal nucleus; sc, superior colliculus; scp, superior cerebellar peduncle; SFi, septofimbrial nucleus; tfp, transverse fibers of pons; Th, thalamus; V1, primary visual cortex; V2, secondary visual cortex; VP, ventral pallidum; VTA, ventral tegmental area.

We used group ICA to retrieve resting-state networks from rs-fMRI data collected from 2-week-old naïve rats (PD13–17; *N* = 11; 5 female and 6 male from 4 different litters) under light anesthesia. We report a total of eight resting-state networks in the infant rat brain. Complete spatial maps data of identified resting-state networks in 2-week-old rats are shown in Supplemental Table 1. Representative spatial maps of all identified resting-state networks are illustrated in Figure 4. We show that at this early age, neuro-anatomical networks are organized as spontaneous fluctuations within a network in the absence of any stimuli. Specifically, the first seven out of eight identified networks (Figure 4; see also Supplemental Table 1) corresponded to seven previously published networks in the adult rats (Becerra et al., 2011), five of which were spatially correlated with Pearson's *r* (see Table 1). The last network did not correlate with an adult template network but showed biologically relevant activity (see below). Specifically, we report the following networks:

#### Default mode network

Two components were consistent with the default mode network. Anterior component (Figure 4-1A) included rostral structures of the anterior forebrain (primary and secondary motor cortex, primary somatosensory cortex, insular cortex, corpus callosum), as well as several subcortical structures (anterior olfactory nucleus, claustrum, caudate putamen). Posterior component (Figure 4-1B) included caudal structures of the cortex (retrosplenial cortex, somatosensory cortex, visual cortex), as well as subcortical structures (caudate putamen, thalamus, amygdala, pretectum).

#### Sensory (exteroceptive) network

This network is comprised of three separate components, two of which show anatomical regions of coherent activity in coronal sections proximal to Bregma, while the third involves regions both proximal and distal to Bregma. Proximal components show coherent activity of similar anatomical regions on the left (Figure 4-2A) and right (Figure 4-2B) hemispheres of the forebrain, respectively. These regions include motor cortices, as well as primary somatosensory cortex. The third component (Figure 4-2C) included primary and secondary somatosensory cortex, insular and olfactory cortex, as well as corpus callosum. In addition, subcortical structures of the third component included caudate putamen, extended amygdala, and ventroposterior and posterior thalamic nuclei.

#### Salience (interoceptive) network

This network was comprised of two components that showed coherent activity of similar cortical regions on either left (Figure 4-3A) or the right hemisphere (Figure 4-3B) of the forebrain primarily distal to Bregma. These cortical regions included secondary motor cortex, primary somatosensory cortex, insular cortex, and corpus callosum. One of the components constituted the majority of the subcortical activation that included: ventral pallidum, hippocampus, as well as anterior, posterior and periventricular thalamic nuclei (Figure 4-3A). Second component showed activation in the lateral thalamic nucleus (Figure 4-3B).

#### Basal Ganglia–Thalamic–hippocampal network

Coherent activations corresponding to this network were present in two components. In the first component, connectivity was noted in several cortical areas: motor, olfactory, parietal, as well as somatosensory cortex. Subcortical structures included different regions of caudate-putamen, thalamus, hippocampus, and hypothalamus (Figure 4-4A). The second component (Figure 4-4B) displayed activity in the CA1 region of the hippocampus and some surrounding structures, including the subiculum and retrosplenial cortex.

#### Basal Ganglia network

This network was composed of two components that primarily involved basal ganglia (caudate-putamen) and surrounding structures. The first component (Figure 4-5A) included left lateralized caudate-putamen and a small portion of the primary somatosensory cortex. The second component (Figure 4-5B) was comprised of coherent activity noted in the rostral brain regions such as right lateralized caudate-putamen, nucleus accumbens, ventral pallidum, insular cortex, globus pallidus, and lateral preoptic area. Coherent activity was also noted caudally, in structures of pontine reticular formation and cerebellum (not shown; Supplemental Table 1).

#### Autonomic network

Coherent connectivity was noted most rostrally in caudate-putamen, thalamic nuclei (midline, intralaminar, ventral anterior, ventral posterior, ventral lateral, and posterior nuclei), parts of hippocampal formation, lateral hypothalamus, as well as peri-olfactory cortex (Figure 4-6). Caudally, coherent activity in this component was noted in retrospenial cortex, lateral olfactory cortex, pretectum just anterior to the superior coliculli and above periaqueductal gray, and to a lesser degree within the reticular formation of the brainstem (not shown; Supplemental Table 1).

#### Cerebellar network

This network was composed of a component that primarily involved anatomical structures of the cerebellum (Figure 4-7): anterior cerebellar connectivity of paraflocculus and cerebellar lobules 6 and 7 with several forebrain structures (somatosensory cortex, caudate-putamen, hippocampus, thalamus, amygdala; Supplemental Table 1).

#### Thalamic-brainstem network

In this network, connectivity was observed mostly in subcortical and brainstem regions. All three components of this network showed coherent activity in different regions of the cortex, thalamus, and to a lesser degree basal ganglia. Activity was distributed along the brainstem within each of the components. It included dorsal areas of pretectum and periaqueductal gray in the first component (Figure 4-8A); superior and inferior colliculi, rubral area, reticular formation of midbrain and pons, as well as nucleus raphe magnus in the second component (Figure 4-8B); ventral pons and ventromedial medulla in the third component (Figure 4-8C).

## Discussion

In order to characterize robust resting-state networks in naïve 2-week-old rats, we applied group ICA to rs-fMRI data acquired under light anesthesia with <1% Isoflurane/O_2_ at 1 L/min using 7T MRI scanner. This is the first report to demonstrate rs-fMRI analysis as an effective tool for the identification of specific low-frequency resting-state networks in the infant rat brain.

### Resting-state networks

It is well-described that functional connectivity measured with rs-fMRI reflects frequent long-range neuronal communication processes delineating a set of large-scale networks across the brain (Damoiseaux et al., 2006; Smith et al., 2009). In this report, we mapped a total of eight networks that comprise interacting regions in the developing rat brain. They closely resemble previously described 8 (Hutchison et al., 2010) and 12 (Lu et al., 2012) discrete functional networks in anesthetized and 7 networks in awake (Becerra et al., 2011) adult rats that implemented the same group ICA analysis.

#### Developed networks

Only the Autonomic Network in 2-week-old rats showed high similarity with autonomic networks in anesthetized (Hutchison et al., 2010; Zhang et al., 2010; Becerra et al., 2011) and awake (Hutchison et al., 2010; Zhang et al., 2010; Becerra et al., 2011) adult rats, with connectivity between hypothalamus, hippocampus and several thalamic nuclei. This suggests that the autonomic network may be one of the first brain networks to reach maturity in the rat. This is perhaps not surprising given that diencephalic structures (including the hypothalamus) are employed early in homeostatic function (Van Dijk and Challis, 1989; Levine, 1994). Early synchronization of the autonomic network reflects the necessity of proper autonomic functioning during infancy and may be evolutionarily optimized to ensure survival.

#### Undeveloped networks

The remaining resting-state networks in 2-week-old rats show immaturity (e.g., Cerebellar network), and/or separation of components along the anterior-posterior axis (e.g., Default Mode, Sensory, and Thalamic-Brainstem network), or with respect to laterality [e.g., Salience (Interoceptive), Basal Ganglia-Thalamic-Hippocampal, and Basal Ganglia network; Figure 4]. This is in contrast to an adult rat brain where multiple independent, bilaterally synchronous resting-state networks were documented in cortical and subcortical areas of the rat brain both under Isoflurane anesthesia (Hutchison et al., 2010) and awake (Becerra et al., 2011). The precise composition of components extracted using ICA is dependent upon the preset dimensionality of the analysis (Leech et al., 2012). In other words, the more components ICA extracts the more fragmented the networks become, and vice versa. However, previous studies in anesthetized (Hutchison et al., 2010) and awake (Becerra et al., 2011; Liang et al., 2011) adult rats used the same dimensionality (40 components extracted) for group ICA analysis, as we used in this study. Such approach preserves most of the variance in the resting-state data and provides a manageable number of components for further analysis. This separation of networks into several components was present when our analysis extracted both 40 and 20 (data not shown) components. Furthermore, both anterior (Figure 4-1A) and posterior (Figure 4-1B) components of the default mode network in infant rats showed spatial overlap with the cortical structures of the default mode network identified in anesthetized (Hutchison et al., 2010) and awake (Becerra et al., 2011; Liang et al., 2011) adult rats, the network appeared undeveloped at 2-weeks of age since subcortical structures were not identified at this early age. Being that the default mode network (although not identical) is highly conserved among species (Pawela et al., 2008; Lu et al., 2012; Smucny et al., 2014), it was not a surprise to demonstrate striking similarities in default mode network architecture between the presented study in lightly anesthetized 2-week-old rats and those of anesthetized (Pawela et al., 2008; Hutchison et al., 2010; Lu et al., 2012; Liang et al., 2015) and awake (Zhang et al., 2010; Becerra et al., 2011; Liang et al., 2015) adult rats. For lists of intrinsic networks identified in the adult and developing human brain, see studies by Smith (Smith et al., 2009) and Fransson (Fransson et al., 2007), respectively. Other imaging modalities such as multichannel near-infrared spectroscopy (Homae et al., 2010) showed that connectivity between (1) homologous bilateral structures are decreased in posterior regions and increased in frontal regions throughout the first 6 months of life, while (2) anterior-posterior connectivity displayed a U-shaped developmental trajectory, with decreasing connectivity for the first 3 months and increased connectivity from 3 to 6 months. Using metabolic connectivity analysis, recently published work in rats (Choi et al., 2015) suggested that large-scale functional networks (1) matured to increase anterior-posterior long-distance connections and (2) achieved energetically efficient wiring in the midline structures during maturation from childhood (5-weeks) via adolescence (10-weeks) to early adulthood (15-weeks). Similar studies of ontogeny of resting-state networks using BOLD along the different time points during development in rodent models should also be performed to corroborate these findings. All together, it is possible that the presence of networks with multiple components is reflective of their developmental immaturity and not a result of anesthesia or a high dimensional analysis.

### Development of network architecture

It is known that processes of apoptosis and axonal pruning are considered a refinement of the embryonic nervous system during early postnatal development (Rice and Barone, 2000). Over the past decade, the view has emerged that the response properties of particular brain regions are largely determined not only by its patterns of connectivity to other regions, but also by their current activity states (Friston and Price, 2001). Myelination starts prenatally at different times for different brain regions and continues throughout development all the way into the early adulthood (Asou et al., 1995; Rice and Barone, 2000; Fields, 2005). Importantly, synaptogenesis begins in the cortex at PD10 and ends at PD30 (Rice and Barone, 2000), contributing to increasing connectivity between cells. In addition, there is substantial evidence of activity-dependent plasticity in myelination (for review, see Zalc and Fields, 2000). It is relatively clear that patterns of long-range neuronal networks are shaped by the *structural* (connectional) properties of the brain (Honey et al., 2007; Damoiseaux and Greicius, 2009; van den Heuvel et al., 2009), which has yet to be confirmed during different time points of the rat brain development. The most rapid growth in both total brain volume and white matter from birth to ~4 months is consistent with the emergence of cognitive abilities in macaques at that age (Malkova et al., 2006). Future diffusion tensor imaging studies would provide insight as to how much of the presented findings are attributable to changes in axonal or synaptic density, as apposed to changes in the neurovascular coupling strength. A high degree of *functional* brain architecture or inter-region temporal correlation is remarkably conserved across species (from nematodes to humans) and is characterized by two fundamental characteristics: high clustering and high efficiency in functions between regions (Smucny and Tregellas, 2013). As a result, studies of human functional brain development are based on the assumption that the interactions between brain regions are critical for the development of each one, and that networks of regions give rise to emerging functions as a coherent whole (Johnson, 2001, 2011a,b). As the brain grows and expanding myelination allow for faster information transmission (Menon, 2013), it was proposed that a “small world” architecture (being functionally optimal early in development), in which major cortical hubs are confined to primary sensory and motor areas, gives way to longer distance connectivity between a few major hubs (Sur and Leamey, 2001; Supekar et al., 2013) during development leading to increasingly hierarchical and more developed structure of the brain (Bullmore and Sporns, 2012). Future studies could include the application of graph theoretical analyses to understand network topology. Studies should also be focused on setting up reliable methodologies for the detection of brain networks using EEG data collected during rs-fMRI, removal of artifacts induced by the multimodal imaging approach, and high-precision neuronal source localization (Ganzetti and Mantini, 2013). Thus, presented data of robust intrinsic networks in a 2-week-old rat can serve as a basis for future studies on maturation of brain architecture over several different time points of development from both a structural and functional standpoint. Our better understanding of the network systems during different time points in development can also, in the long term, lead to important advances in our diagnostic, therapeutic, and prognostic capacities of various pediatric brain pathologies.

### Practical applications

A major advantage of task-independent rs-fMRI in animal models is in its translational utility. In addition to accumulating data in humans, important findings have also been generated in mice, rats, and non-human primates that indeed confirmed dynamic functional connectivity in the absence of stimuli as a fundamental property in the mammalian brain. Since the approach does not require for animals to perform a task, animals can either be anesthetized (Hutchison et al., 2010; Magnuson et al., 2010; Wang et al., 2011) or restrained during scanning (Becerra et al., 2011; Hutchison et al., 2013; Liang et al., 2015), providing suitable conditions for analysis of intrinsic networks using methods analogous to those used in human data (Smucny et al., 2014). Similarly to presented data, previous studies in infants showed incomplete versions of networks (Gao et al., 2009; Smyser et al., 2010) suggesting that networks develop in parallel with the ontogeny of stimulus-independent cognitive competencies (Fair et al., 2008; Thomason et al., 2008). Although exact equivalencies cannot be made between the rodent and human developing age, there is a consensus that PD1-14 roughly extends from the last trimester of pregnancy up to the first few years of postnatal life in humans (Huttenlocher and Dabholkar, 1997; Rice and Barone, 2000; Clancy et al., 2001, 2007). Similarities in resting-state networks among species highlight translational utility of the rs-fMRI method in studying developmental disease states in animal models (Jones et al., 2014; Johnson et al., 2015).

### Methodological considerations

#### Anesthesia level

Level of anesthesia can affect the BOLD signal and subsequently alter detection of resting-state networks as demonstrated in adult rat model (Wang et al., 2011; Liang et al., 2015). Although under higher levels of anesthesia (>2% Isoflurane) brain networks showed decreased or no connectivity (Wang et al., 2011; Liang et al., 2015), adequate anesthesia (<1.5% Isoflurane) leads to a good correlation between cerebral perfusion (Magnuson et al., 2010), electrical activity produced by the brain (Liu et al., 2011; Liang et al., 2015), and the BOLD signal (Wang et al., 2011). The use of anesthesia in adult rodent neuroimaging and the inherent limitations have been extensively described elsewhere (Becerra et al., 2011; Thompson and Bushnell, 2012; D'Souza et al., 2014; Jonckers et al., 2014). We used respiratory rate as a useful physiological index to gauge the depth of anesthesia. When awake, adult rats exhibit low regular breathing, also described as the dominant respiratory rate reported to be in a range of 60–100 breaths/min (Kabir et al., 2010; Carnevali et al., 2013). Our preliminary data in adult rats indicate that the light levels of anesthesia (1–1.5% Isoflurane/O_2_ at 1 L/min) during functional scanning are associated with respiratory rate in the range of 49–55 breaths/min (Bajic and Becerra, unpublished observations). However, for 2-week-old rats, the anesthesia requirement was lower. Unlike the constant anesthesia requirement in adult rodents (Eger and Johnson, 1987), the anesthesia requirement in infant rodents rather decreases steadily over time with increasing duration of anesthesia administration (Stratmann et al., 2009; Kodama et al., 2011). To maintain respiratory rate in a narrow range of 45–50 breaths/min (Figures 1B,C), 2-week-old rats received steady and uniform anesthetic (<1% Isoflurane/O_2_ at 1 L/min) during 10 min of functional scanning. Such minimal anesthesia requirement allowed for adequate sedation associated with immobility, resulting in detection of the resting-state networks at this early age.

#### Registration of 2-week-old rat brain images to an adult rat brain template

Not only is the cortical surface area smooth in the rat brain, irrespective of the age, but also the difference in size between 2-week-old and adult rat brain is minimal (measured in mm). In contrast to the availability of detailed adult rat standard brain atlases (Konig and Klippel, 1963; Pelligrino et al., 1979; Paxinos and Watson, 1998; Swanson, 2003), no comprehensive atlases are available for the infant rat brain (Ramachandra and Subramanian, 2011). Instead of creating a new atlas, we provided evidence of appropriate registration for infant rats at 2-weeks of age with our adult rat template (Figure 2). Furthermore, registration with adult templates would be advantageous for follow up or longitudinal studies within the same or different animals when studying network development or long-term drug effects.

## Conclusion

We provide evidence that resting-state networks in 2-week-old rats are detectable and closely resemble template networks of the adult rat. Resting-state networks are preserved under pharmacologically induced light anesthesia and are highly reproducible in infant rats. These empirical findings will be important for conducting studies that aim at discerning the topological organization of resting-state networks during development and in various models of developing rat brain disorders.

## Author contributions

Authorship credit was based on substantial contributions to (1) the conception and study design (DBa, DBo, LB); (2) acquisition and analysis of data, or interpretation of data (DBa, MC, LB); (3) drafting the article or revising it critically for important intellectual content (DBa, MC, LB); (4) final approval of the version to be published (DBa, MC, DBo, LB); and (5) are accountable for all aspects of the work in ensuring that questions related to the accuracy or integrity of any part of the work are appropriately investigated and resolved (DBa, MC, DBo, LB).

### Conflict of interest statement

The authors declare that the research was conducted in the absence of any commercial or financial relationships that could be construed as a potential conflict of interest.

## Abbreviations

BOLD: blood oxygen level-dependent
EPI: echo planar imaging
ICA: independent component analysis
MRI: magnetic resonance imaging
PD: postnatal day
rs-fMRI: resting-state functional magnetic resonance imaging
TE: echo time
TR: repetition time.
