# Supplementary material for: Probing Intrinsic Resting-State Networks in the Infant Rat Brain
Source: Front Behav Neurosci. 2016 Oct 18;10:192. doi: 10.3389/fnbeh.2016.00192 (PMC5067436; doi:10.3389/fnbeh.2016.00192)
Supplement: Supplementary file 1 [file Table1.DOCX]

**TABLE 1**

**Resting-State Networks in 2-Week Old Rat**

| **1A. Default Mode Network - Anterior Component** | | | | | | |
| --- | --- | --- | --- | --- | --- | --- |
| **Brain Region** | **Laterality** | **z-stat** | **X(mm)** | **Y(mm)** | **Z(mm)** | **Vol (mm^3^)** |
| **Cortical Structures** |  |  |  |  |  |  |
| Motor Cortex Primary | R | 4.97 | 3.28 | 5.74 | 3.00 | 3.57 |
|  | L | 3.73 | -3.87 | 4.80 | 3.00 | 2.98 |
| Somatosensory Cortex Primary Jaw Region | L | 3.26 | -4.69 | 5.27 | 2.00 | 1.29 |
| Olfactory Cortex Lateral | L | 3.95 | -3.87 | 2.46 | 1.00 | 0.80 |
|  | L | 2.30 | -6.33 | 2.11 | -5.00 | 1.06 |
|  | R | 2.62 | 3.87 | 5.27 | -9.00 | 0.81 |
| Cingulate Cortex | R | 3.88 | 0.23 | 6.09 | 0.00 | 2.87 |
| Somatosensory Cortex Primary Forelimb Region | L | 2.35 | -4.22 | 8.09 | 0.00 | 1.32 |
|  | L | 2.25 | -3.40 | 8.44 | -1.00 | 1.11 |
| Somatosensory Cortex Primary Hindlimb Region | R | 8.04 | 1.76 | 8.09 | -1.00 | 148.82 |
|  | L | 2.81 | -1.87 | 9.26 | -2.00 | 1.83 |
| Insular Cortex | L | 3.32 | -5.86 | 2.93 | -1.00 | 2.14 |
| Somatosensory Cortex Secondary | R | 2.43 | 5.39 | 4.45 | -1.00 | 0.52 |
| Retrosplenial Cortex | L | 4.17 | -0.23 | 8.09 | -3.00 | 0.52 |
|  | L | 3.96 | -0.59 | 7.73 | -3.00 | 0.60 |
|  | L | 2.80 | -1.52 | 8.91 | -7.00 | 0.81 |
| Somatosensory Cortex Primary Barrel Field | L | 2.87 | -3.87 | 7.73 | -3.00 | 1.24 |
| Corpus Callosum | R | 2.80 | 4.92 | 4.45 | -4.00 | 1.46 |
| Somatosensory Cortex Primary Trunk Region | R | 2.68 | 2.23 | 8.44 | -4.00 | 0.70 |
| Visual Cortex Secondary | R | 2.35 | 3.75 | 8.09 | -6.00 | 1.62 |
|  | L | 2.06 | -4.57 | 7.73 | -7.00 | 0.51 |
|  | R | 3.40 | 4.57 | 6.56 | -9.00 | 4.81 |
| Perirhinal Cortex | R | 4.77 | 5.04 | 4.10 | -8.00 | 5.97 |
| **Subcortical Structures** |  |  |  |  |  |  |
| Olfactory Nucleus Anterior | L | 5.07 | -1.05 | 2.11 | 3.00 | 4.42 |
| Claustrum | L | 3.65 | -2.23 | 4.92 | 3.00 | 1.70 |
| Striatum Ventral | L | 2.99 | -1.05 | 2.93 | 2.00 | 0.52 |
| Caudate & Putamen | L | 6.05 | -3.52 | 3.28 | 0.00 | 1.77 |
|  | L | 4.69 | -4.22 | 2.81 | 0.00 | 1.13 |
|  | L | 5.64 | -3.05 | 4.45 | -1.00 | 1.65 |
| Septal Region Medial Group | L | 2.50 | -0.23 | 4.10 | 0.00 | 0.47 |
|  | R | 2.25 | 0.12 | 3.75 | 0.00 | 0.44 |
| Extended Amygdala Medial Division | R | 3.11 | 1.06 | 3.28 | -1.00 | 1.50 |
| Hippocampal Formation CA1 Field | L | 3.11 | -0.70 | 6.09 | -3.00 | 0.85 |
|  | L | 2.75 | -4.69 | 6.80 | -5.00 | 1.90 |
|  | L | 2.09 | -3.87 | 1.64 | -6.00 | 1.00 |
| Amygdaloid Nuclear Complex Laterobasal | L | 3.55 | -5.51 | 1.99 | -3.00 | 3.52 |
| Corticospinal Tract | L | 2.51 | -4.34 | 5.27 | -3.00 | 0.60 |
|  | R | 3.21 | 3.75 | 3.75 | -3.00 | 2.65 |
| Fimbria of Hippocampus | L | 3.47 | -2.69 | 7.73 | -4.00 | 2.21 |
|  | R | 2.32 | 3.28 | 7.38 | -5.00 | 0.44 |
| Hippocampal Formation CA3 Field | L | 3.23 | -4.69 | 2.93 | -5.00 | 1.44 |
|  | R | 1.94 | 3.75 | 4.92 | -6.00 | 0.37 |
| Hypothalamus Medial Zone | L | 3.11 | -0.70 | 2.46 | -5.00 | 0.63 |
| Hippocampal Formation Dentate Gyrus | L | 4.16 | -4.22 | 5.62 | -7.00 | 0.96 |
| Hippocampal Formation Subicular Complex | L | 4.31 | -4.22 | 5.27 | -8.00 | 1.35 |
| **Brainstem/Cerebellar Structures** |  |  |  |  |  |  |
| Cerebellar Peduncle | L | 3.90 | -2.69 | 0.94 | -8.00 | 1.57 |
| Trigeminal Nerve | R | 3.35 | 2.58 | 1.29 | -9.00 | 2.31 |
| Inferior Colliculus | L | 2.15 | -2.23 | 6.09 | -9.00 | 0.38 |
| Cerebellum Lobule 2 | L | 2.66 | -1.05 | 5.27 | -10.00 | 0.89 |
|  |  |  |  |  |  |  |
| **1B. Default Mode Network - Posterior Component** | | | | | | |
| **Brain Region** | **Laterality** | **z-stat** | **X(mm)** | **Y(mm)** | **Z(mm)** | **Vol (mm^3^)** |
| **Cortical Structures** |  |  |  |  |  |  |
| Motor Cortex Secondary | R | 3.67 | 1.06 | 8.09 | 3.00 | 2.38 |
|  | L | 6.64 | -0.70 | 8.09 | -1.00 | 5.69 |
| Cingulate Cortex | R | 4.25 | 0.12 | 7.73 | 2.00 | 1.32 |
| Motor Cortex Primary | L | 3.53 | -1.87 | 7.27 | 0.00 | 1.18 |
| Corpus Callosum | R | 5.59 | 3.40 | 6.09 | -1.00 | 1.69 |
|  | L | 4.13 | -2.69 | 8.09 | -7.00 | 0.69 |
| Insular Cortex | R | 5.51 | 4.57 | 2.11 | -1.00 | 0.85 |
| Retrosplenial Cortex | R | 6.34 | 0.59 | 7.73 | -2.00 | 2.46 |
|  | R | 5.80 | 0.59 | 8.09 | -3.00 | 1.74 |
|  | R | 6.68 | 0.47 | 8.44 | -6.00 | 3.41 |
|  | R | 6.56 | 0.12 | 8.44 | -7.00 | 0.48 |
| Somatosensory Cortex Primary Trunk Region | L | 8.15 | -2.23 | 8.44 | -4.00 | 2.79 |
|  | L | 7.66 | -1.87 | 8.09 | -4.00 | 1.40 |
| Somatosensory Cortex Primary | L | 3.43 | -5.86 | 7.27 | -5.00 | 0.96 |
| Visual Cortex Secondary | L | 6.50 | -2.23 | 8.55 | -6.00 | 6.19 |
|  | L | 3.99 | -5.04 | 8.55 | -6.00 | 0.47 |
| Visual Cortex Primary | L | 3.87 | -3.87 | 8.44 | -7.00 | 0.37 |
|  | L | 6.21 | -2.69 | 8.91 | -9.00 | 1.90 |
| **Subcortical Structures** |  |  |  |  |  |  |
| Caudate & Putamen | L | 6.24 | -4.22 | 4.45 | 1.00 | 1.04 |
|  | L | 4.23 | -4.69 | 3.63 | -2.00 | 3.09 |
| Thalamus Mediodorsal Nucleus | L | 3.90 | -0.70 | 4.92 | -3.00 | 0.73 |
|  | R | 4.91 | 0.94 | 4.10 | -4.00 | 2.07 |
| Extended Amygdala Central Division | L | 3.45 | -4.69 | 2.46 | -3.00 | 0.63 |
| **Brainstem/Cerebellar Structures** |  |  |  |  |  |  |
| Pretectum | R | 3.91 | 0.59 | 5.27 | -5.00 | 0.92 |
| Tegmental Area Ventral | R | 4.08 | 0.12 | 2.11 | -7.00 | 0.63 |
| Cerebellum Lobule 4 & 5 | R | 4.35 | 2.58 | 5.27 | -10.00 | 1.17 |
| Cerebellar Peduncle | R | 4.03 | 2.58 | 3.75 | -10.00 | 0.93 |
|  |  |  |  |  |  |  |

| **2A. Sensory (Exteroceptive) Network - Component 1** | | | | | | |
| --- | --- | --- | --- | --- | --- | --- |
| **Brain Region** | **Laterality** | **z-stat** | **X(mm)** | **Y(mm)** | **Z(mm)** | **Vol (mm^3^)** |
| **Cortical Structures** |  |  |  |  |  |  |
| Motor Cortex Secondary | L | 7.44 | -2.69 | 7.73 | 4.00 | 2.83 |
|  | R | 4.61 | 2.11 | 8.09 | 4.00 | 0.93 |
| Orbitofrontal Cortex | L | 5.63 | -2.23 | 5.74 | 4.00 | 5.45 |
| Motor Cortex Primary | L | 6.98 | -3.87 | 4.92 | 3.00 | 19.82 |
|  | L | 9.34 | -2.23 | 7.62 | 2.00 | 7.20 |
| Somatosensory Cortex Primary Upper Lip Region | R | 5.87 | 5.27 | 6.09 | 0.00 | 1.10 |
|  | L | 4.12 | -4.22 | 6.09 | 0.00 | 1.51 |
| Somatosensory Cortex Primary Barrel Field | R | 7.83 | 4.22 | 6.91 | -1.00 | 2.14 |
|  | R | 6.53 | 4.92 | 6.44 | -1.00 | 0.78 |
|  | L | 4.54 | -5.86 | 6.56 | -1.00 | 2.27 |
| Somatosensory Cortex Secondary | L | 7.41 | -6.68 | 5.27 | -2.00 | 1.19 |
|  | L | 6.93 | -6.33 | 4.57 | -2.00 | 4.67 |
| Somatosensory Cortex Primary Dysgranular Region | L | 7.20 | -3.40 | 8.09 | -3.00 | 1.15 |
| Auditory Cortex Primary | R | 4.11 | 5.39 | 4.10 | -5.00 | 0.40 |
| Perirhinal Cortex | L | 5.27 | -6.56 | 3.75 | -8.00 | 2.47 |
| Corpus Callosum | R | 4.28 | 3.75 | 6.44 | -8.00 | 0.80 |
| **Brainstem/Cerebellar Structures** |  |  |  |  |  |  |
| Inferior Colliculus | R | 4.49 | 0.94 | 6.91 | -9.00 | 1.79 |
| **2B. Sensory (Exteroceptive) Network - Component 2** | | | | | | |
| **Brain Region** | **Laterality** | **z-stat** | **X(mm)** | **Y(mm)** | **Z(mm)** | **Vol (mm^3^)** |
| **Cortical Structures** |  |  |  |  |  |  |
| Orbitofrontal Cortex | L | 3.17 | -0.23 | 4.45 | 4.00 | 2.47 |
| Somatosensory Cortex Primary Jaw Region | R | 9.02 | 3.40 | 5.74 | 2.00 | 86.61 |
|  | L | 6.15 | -4.69 | 6.91 | 1.00 | 13.09 |
| Olfactory Cortex Lateral | R | 2.68 | 2.58 | 1.99 | 2.00 | 0.80 |
|  | L | 3.02 | -6.33 | 1.99 | -5.00 | 0.85 |
| Somatosensory Cortex Secondary | L | 4.62 | -6.68 | 3.63 | -2.00 | 1.74 |
| Insular Cortex | L | 4.37 | -6.21 | 3.28 | -2.00 | 0.71 |
|  | L | 5.15 | -5.86 | 4.80 | -3.00 | 4.00 |
| Corpus Callosum | L | 4.21 | -1.05 | 6.91 | -3.00 | 1.70 |
|  | R | 2.75 | 4.92 | 5.27 | -5.00 | 0.96 |
|  | R | 3.74 | 2.93 | 7.27 | -7.00 | 6.06 |
| Somatosensory Cortex Primary Barrel Field | R | 3.75 | 5.39 | 6.56 | -3.00 | 1.03 |
|  | L | 3.49 | -6.21 | 6.91 | -3.00 | 1.48 |
| Perirhinal Cortex | L | 4.15 | -7.03 | 3.28 | -7.00 | 1.32 |
| Retrosplenial Cortex | L | 2.99 | -1.41 | 7.62 | -7.00 | 1.11 |
| Visual Cortex Secondary | L | 2.40 | -4.69 | 6.80 | -8.00 | 0.40 |
| **Subcortical Structures** |  |  |  |  |  |  |
| Septal Region Lateral Group | R | 2.72 | 0.12 | 3.75 | 1.00 | 0.73 |
| Cingulum | L | 3.26 | -1.87 | 6.91 | -1.00 | 0.70 |
| Extended Amygdala Central Division | R | 2.68 | 1.29 | 2.93 | -1.00 | 0.84 |
| Olfactory Amygdala | L | 3.05 | -3.52 | 0.12 | -2.00 | 1.58 |
| Hippocampal Formation Dentate Gyrus | L | 3.43 | -0.70 | 5.74 | -3.00 | 1.13 |
|  | R | 4.58 | 3.75 | 2.81 | -6.00 | 1.48 |
| Zona incerta | R | 3.42 | 1.76 | 2.46 | -3.00 | 0.98 |
| Thalamus Lateral Nucleus | R | 5.20 | 1.76 | 5.27 | -5.00 | 3.05 |
| Hippocampal Formation CA1 Field | L | 4.37 | -2.23 | 7.27 | -5.00 | 2.94 |
|  | L | 2.91 | -3.05 | 7.73 | -5.00 | 1.00 |
| Hippocampal Formation Subicular Complex | L | 3.37 | -3.05 | 6.91 | -7.00 | 1.83 |
| **Brainstem/Cerebellar Structures** |  |  |  |  |  |  |
| Superior Colliculus | L | 3.21 | -0.23 | 6.91 | -8.00 | 2.07 |
| Trigeminal Nerve | L | 2.53 | -3.40 | 1.29 | -8.00 | 1.87 |
|  | R | 2.90 | 1.76 | 1.29 | -9.00 | 1.25 |
| Inferior Colliculus | L | 4.94 | -1.05 | 6.44 | -9.00 | 1.51 |
|  | R | 4.74 | 1.76 | 6.09 | -10.00 | 2.90 |
| Cerebellum Lobule 4 & 5 | R | 5.40 | 2.58 | 5.27 | -10.00 | 1.58 |
|  | R | 5.36 | 2.23 | 5.62 | -10.00 | 1.15 |
| Simple Lobule | R | 4.92 | 3.40 | 5.62 | -11.00 | 9.57 |
|  | L | 2.82 | -4.57 | 6.80 | -11.00 | 0.56 |
| Cerebellum Lobule 5 | L | 3.13 | -0.70 | 8.91 | -11.00 | 0.69 |

| **2C. Sensory (Exteroceptive) Network - Component 3** | | | | | | |
| --- | --- | --- | --- | --- | --- | --- |
| **Brain Region** | **Laterality** | **z-stat** | **X(mm)** | **Y(mm)** | **Z(mm)** | **Vol (mm^3^)** |
| **Cortical Structures** |  |  |  |  |  |  |
| Motor Cortex Secondary | L | 3.54 | -1.87 | 7.38 | 4.00 | 0.66 |
| Orbitofrontal Cortex | R | 2.68 | 0.94 | 5.27 | 4.00 | 0.38 |
|  | L | 3.87 | -2.69 | 4.10 | 3.00 | 0.77 |
|  | R | 3.20 | 1.76 | 4.10 | 3.00 | 0.44 |
| Insular Cortex | R | 3.70 | 3.75 | 3.75 | 2.00 | 1.14 |
| Motor Cortex Primary | L | 3.96 | -2.69 | 8.09 | 2.00 | 4.17 |
| Olfactory Cortex Lateral | L | 4.19 | -5.04 | 2.11 | -1.00 | 3.02 |
|  | R | 5.89 | 4.57 | 0.12 | -4.00 | 3.02 |
|  | R | 2.75 | 3.40 | 3.28 | -9.00 | 0.62 |
| Somatosensory Cortex Primary Barrel Field | R | 7.42 | 4.92 | 6.91 | -2.00 | 55.14 |
|  | L | 5.06 | -4.22 | 7.38 | -3.00 | 5.51 |
| Somatosensory Cortex Primary Forelimb Region | L | 3.17 | -2.69 | 8.91 | -2.00 | 1.02 |
| Somatosensory Cortex Primary Trunk Region | L | 3.00 | -3.40 | 9.26 | -4.00 | 0.66 |
| Somatosensory Cortex Primary | L | 7.84 | -5.86 | 6.91 | -5.00 | 3.54 |
| Parietal Cortex Posterior Area | R | 3.49 | 3.28 | 8.91 | -5.00 | 1.22 |
|  | L | 2.94 | -4.57 | 8.44 | -5.00 | 0.63 |
| Visual Cortex Secondary | L | 3.58 | -4.57 | 7.73 | -6.00 | 1.92 |
|  | L | 2.96 | -5.04 | 7.73 | -7.00 | 0.38 |
| Temporal Association Cortex | R | 4.61 | 4.92 | 4.92 | -8.00 | 0.85 |
| **Subcortical Structures** |  |  |  |  |  |  |
| Caudate & Putamen | L | 3.87 | -3.87 | 4.92 | 1.00 | 1.25 |
|  | L | 3.14 | -3.87 | 3.28 | 0.00 | 1.73 |
| Extended Amygdala Medial Division | R | 4.02 | 2.58 | 1.29 | -3.00 | 1.11 |
| Thalamus Ventral Posterior Complex | L | 3.67 | -3.52 | 4.92 | -3.00 | 0.71 |
|  | L | 4.68 | -3.40 | 3.75 | -3.00 | 5.78 |
| Thalamus Posterior Nucleus | R | 3.50 | 1.41 | 4.10 | -3.00 | 1.10 |
| Corticospinal Tract | L | 3.39 | -4.22 | 5.74 | -3.00 | 0.48 |
| Auditory Thalamus | R | 3.64 | 2.46 | 4.45 | -6.00 | 0.62 |
| Hippocampal Formation Dentate Gyrus | R | 3.67 | 3.40 | 4.80 | -7.00 | 1.19 |
| Hippocampal Formation Subicular Complex | R | 3.76 | 3.75 | 4.10 | -8.00 | 0.85 |
| **Brainstem/Cerebellar Structures** |  |  |  |  |  |  |
| Optic Nerve | R | 3.84 | 2.93 | 2.11 | -4.00 | 0.89 |
| Dorsal Column | R | 3.17 | 1.41 | 3.28 | -5.00 | 1.10 |
| Rubral Area | R | 2.93 | 0.94 | 3.28 | -6.00 | 0.62 |
| Superior Colliculus | L | 3.77 | -2.69 | 4.80 | -7.00 | 1.83 |
| Substantia Nigra | R | 3.70 | 0.94 | 1.64 | -7.00 | 1.17 |
| Reticular Formation Midbrain | L | 3.46 | -3.05 | 4.10 | -7.00 | 0.84 |
| Subbrachial Nucleus | L | 3.26 | -3.75 | 3.75 | -7.00 | 0.62 |
| Basilar Pontine Nuclei | R | 3.22 | 0.94 | 0.47 | -8.00 | 1.87 |
| Tegmental Nucleus Microcellular | L | 2.84 | -3.05 | 3.28 | -8.00 | 0.47 |
| Trigeminal Nerve | R | 3.16 | 2.58 | 1.29 | -9.00 | 0.76 |
|  | L | 5.94 | -3.05 | 0.94 | -11.00 | 3.34 |
| Periaqueductal Gray Dorsolateral Zone | L | 3.09 | -1.05 | 5.74 | -9.00 | 0.76 |
| Cerebellum Lobule 2 | R | 4.91 | 0.59 | 4.80 | -10.00 | 0.91 |
| Simple Lobule | L | 4.07 | -4.22 | 5.27 | -11.00 | 0.38 |
|  | | | | | | |
| **3A. Salience (Interoceptive) Network - Component 1** | | | | | | |
| **Brain Region** | **Laterality** | **z-stat** | **X(mm)** | **Y(mm)** | **Z(mm)** | **Vol (mm^3^)** |
| **Cortical Structures** |  |  |  |  |  |  |
| Motor Cortex Secondary | L | 3.17 | -1.87 | 8.09 | 4.00 | 1.04 |
|  | L | 2.77 | -3.05 | 6.91 | 4.00 | 0.77 |
|  | R | 2.85 | 0.94 | 7.27 | -1.00 | 0.38 |
| Olfactory Cortex Medial | R | 3.80 | 2.23 | 0.47 | 0.00 | 0.93 |
| Somatosensory Cortex Primary Hindlimb Region | R | 2.94 | 1.76 | 7.62 | -1.00 | 2.14 |
| Somatosensory Cortex Primary Barrel Field | R | 4.28 | 3.75 | 7.38 | -2.00 | 1.55 |
| Olfactory Cortex Lateral | R | 4.04 | 4.22 | 0.47 | -2.00 | 2.93 |
|  | R | 4.07 | 5.04 | 1.29 | -3.00 | 3.47 |
| Somatosensory Cortex Primary Forelimb Region | R | 3.20 | 2.93 | 8.09 | -2.00 | 0.71 |
| Somatosensory Cortex Primary Trunk Region | L | 9.33 | -2.23 | 8.91 | -3.00 | 100.17 |
| Insular Cortex | R | 4.68 | 5.74 | 3.63 | -3.00 | 2.07 |
| Parietal Cortex Posterior Area | L | 3.87 | -4.22 | 8.91 | -5.00 | 0.84 |
| Somatosensory Cortex Primary | R | 3.79 | 5.39 | 6.80 | -5.00 | 0.85 |
| Temporal Association Cortex | L | 4.06 | -7.03 | 4.57 | -6.00 | 1.55 |
| Auditory Cortex Primary | L | 3.59 | -6.68 | 5.39 | -6.00 | 0.55 |
| Visual Cortex Primary | L | 5.03 | -3.05 | 8.09 | -7.00 | 0.70 |
|  | L | 4.04 | -3.87 | 7.27 | -9.00 | 1.36 |
| Corpus Callosum | L | 3.62 | -5.04 | 6.91 | -7.00 | 0.82 |
| **Subcortical Structures** |  |  |  |  |  |  |
| Ventral Pallidum | R | 3.52 | 2.58 | 1.29 | -1.00 | 0.71 |
| Hypothalamus Periventicular Zone | L | 3.18 | -0.70 | 2.11 | -1.00 | 0.74 |
| Fimbria of Hippocampus | L | 3.48 | -1.52 | 6.09 | -2.00 | 0.55 |
| Thalamus Anterior Nuclei | L | 4.02 | -0.59 | 3.75 | -2.00 | 3.61 |
| Thalamus Lateral Nucleus | R | 4.50 | 2.11 | 5.74 | -3.00 | 0.78 |
| Pretectum | R | 2.68 | 1.76 | 6.09 | -6.00 | 0.56 |
| **Brainstem/Cerebellar Structures** |  |  |  |  |  |  |
| Trigeminal Nerve | L | 3.80 | -3.52 | 1.64 | -9.00 | 1.66 |
| Inferior Colliculus | R | 3.11 | 0.12 | 7.27 | -9.00 | 0.84 |
| Reticular Formation Midbrain | L | 3.67 | -2.23 | 4.10 | -9.00 | 1.14 |
| Central Gray of Pons | L | 7.18 | -0.23 | 2.93 | -11.00 | 10.64 |
|  |  |  |  |  |  |  |
| **3B. Salience (Interoceptive) Network - Component 2** | | | | | | |
| **Brain Region** | **Laterality** | **z-stat** | **X(mm)** | **Y(mm)** | **Z(mm)** | **Vol (mm^3^)** |
| **Cortical Structures** |  |  |  |  |  |  |
| Corpus Callosum | L | 5.85 | -4.22 | 6.09 | -1.00 | 0.65 |
| Somatosensory Cortex Primary | R | 9.86 | 5.39 | 5.27 | -2.00 | 28.56 |
|  | L | 5.49 | -6.21 | 6.56 | -2.00 | 1.72 |
| **Subcortical Structures** |  |  |  |  |  |  |
| Thalamus Lateral Nucleus | R | 7.40 | 3.40 | 4.92 | -4.00 | 1.77 |
| **Brainstem/Cerebellar Structures** |  |  |  |  |  |  |
| Superior Colliculus | L | 5.21 | -2.23 | 5.74 | -7.00 | 0.47 |
| Raphe Nuclei Midbrain | L | 8.75 | -0.23 | 2.11 | -9.00 | 1.96 |

|  |  |  |  |  |  |  |
| --- | --- | --- | --- | --- | --- | --- |
| **4A. Basal Ganglia-Thalamic-Hippocampal Network – Component 1** | | | | | | |
| **Brain Region** | **Laterality** | **z-stat** | **X(mm)** | **Y(mm)** | **Z(mm)** | **Vol (mm^3^)** |
| **Cortical Structures** |  |  |  |  |  |  |
| Motor Cortex Secondary | R | 3.08 | 0.94 | 7.62 | 3.00 | 1.11 |
|  | R | 4.14 | 1.41 | 8.09 | 2.00 | 1.36 |
| Motor Cortex Primary | L | 3.43 | -1.87 | 8.09 | 2.00 | 0.41 |
| Corpus Callosum | R | 3.76 | 0.94 | 5.74 | 1.00 | 0.74 |
|  | R | 4.41 | 4.92 | 5.74 | -7.00 | 3.50 |
| Olfactory Cortex Lateral | R | 3.36 | 3.05 | 0.94 | 0.00 | 2.65 |
| Somatosensory Cortex Primary Barrel Field | R | 4.52 | 4.22 | 8.09 | -4.00 | 1.39 |
| Parietal Cortex Posterior Area | R | 4.45 | 3.75 | 8.09 | -5.00 | 1.25 |
| Somatosensory Cortex Primary | R | 3.33 | 4.92 | 7.27 | -5.00 | 1.28 |
| Retrosplenial Cortex | L | 3.24 | -1.05 | 7.62 | -7.00 | 0.38 |
| **Subcortical Structures** |  |  |  |  |  |  |
| Septal Region Lateral Group | L | 6.05 | -1.05 | 4.10 | 0.00 | 4.38 |
| Hypothalamus Lateral Zone | R | 5.29 | 1.29 | 1.76 | -1.00 | 6.19 |
|  | R | 3.24 | 1.29 | 1.29 | -3.00 | 0.41 |
| Septal Region Medial Group | L | 4.30 | -2.23 | 1.29 | -1.00 | 4.12 |
| Hypothalamus Medial Zone | L | 3.17 | -0.70 | 1.29 | -1.00 | 0.76 |
| Caudate & Putamen | R | 3.79 | 3.05 | 3.28 | -1.00 | 2.60 |
|  | L | 6.64 | -3.40 | 5.62 | -2.00 | 44.41 |
| Thalamus Ventral Medial Nucleus | L | 3.29 | -1.41 | 2.46 | -3.00 | 0.67 |
| Thalamus Midline Nuclei | L | 2.97 | -0.23 | 4.45 | -3.00 | 0.38 |
| Hippocampal Formation CA3 Field | L | 4.05 | -3.87 | 2.11 | -5.00 | 0.80 |
| Auditory Thalamus | R | 3.21 | 2.93 | 4.57 | -7.00 | 0.41 |
| Hippocampal Formation Subicular Complex | R | 4.36 | 3.75 | 4.92 | -8.00 | 2.14 |
| **Brainstem/Cerebellar Structures** |  |  |  |  |  |  |
| Auditory Radiation | L | 4.35 | -3.87 | 4.10 | -5.00 | 0.96 |
| Darkschewitch Nucleus | L | 3.58 | -0.23 | 4.10 | -6.00 | 0.77 |
| Nucleus of the Lateral Lemniscus | R | 3.35 | 1.76 | 3.63 | -9.00 | 0.82 |
| Paraflocculus | R | 4.18 | 3.40 | 4.10 | -10.00 | 1.95 |
| Reticular Formation Pontomedullary | R | 3.82 | 0.59 | 3.63 | -10.00 | 1.14 |
| Cerebellum Lobule 3 | R | 3.89 | 0.94 | 6.44 | -11.00 | 1.66 |
| **4B. Basal Ganglia-Thalamic-Hippocampal Network – Component 2** | | | |  |  |  |
| **Brain Region** | **Laterality** | **z-stat** | **X(mm)** | **Y(mm)** | **Z(mm)** | **Vol (mm^3^)** |
| **Cortical Structures** |  |  |  |  |  |  |
| Retrosplenial Cortex | R | 6.06 | 0.59 | 7.27 | -3.00 | 1.18 |
| Somatosensory Cortex Primary Barrel Field | R | 5.56 | 3.28 | 6.91 | -3.00 | 2.62 |
|  | R | 5.52 | 3.40 | 7.62 | -3.00 | 0.89 |
| **Subcortical Structures** |  |  |  |  |  |  |
| Caudate Putamen | R | 6.07 | 1.29 | 4.10 | 2.00 | 0.62 |
| Hippocampal Formation CA1 Field | R | 6.96 | 0.94 | 6.44 | -3.00 | 4.88 |
|  | R | 7.92 | 1.41 | 7.27 | -5.00 | 3.80 |
|  | L | 5.69 | -1.41 | 6.56 | -5.00 | 0.44 |
| Hippocampal Formation Dentate Gyrus | R | 7.18 | 1.76 | 6.09 | -4.00 | 2.73 |
| Fimbria-Fronix | R | 4.50 | 2.93 | 7.62 | -5.00 | 0.59 |
| Hippocampal Formation Dentate Gyrus | R | 5.90 | 3.75 | 4.92 | -7.00 | 1.08 |
|  |  |  |  |  |  |  |
|  |  |  |  |  |  |  |

| **5A. Basal Ganglia Network - Component 1** |  |  |  |  |  |  |
| --- | --- | --- | --- | --- | --- | --- |
| **Brain Region** | **Laterality** | **z-stat** | **X(mm)** | **Y(mm)** | **Z(mm)** | **Vol (mm^3^)** |
| **Cortical Structures** |  |  |  |  |  |  |
| Somatosensory Cortex Primary Jaw Region | L | 11.58 | -3.87 | 5.27 | 2.00 | 2.53 |
|  | L | 10.57 | -4.22 | 4.92 | 2.00 | 3.09 |
| **Subcortical Structures** |  |  |  |  |  |  |
| Caudate & Putamen | L | 11.93 | -3.05 | 4.92 | 1.00 | 10.08 |

| **5B. Basal Ganglia Network - Component 2** | |  |  |  |  |  |
| --- | --- | --- | --- | --- | --- | --- |
| **Brain Region** | **Laterality** | **z-stat** | **X(mm)** | **Y(mm)** | **Z(mm)** | **Vol (mm^3^)** |
| **Cortical Structures** | |  |  |  |  |  |
| Orbitofrontal Cortex | L | 7.90 | -1.41 | 4.10 | 3.00 | 5.64 |
| **Subcortical Structures** | |  |  |  |  |  |
| Striatum Ventral | L | 7.08 | -1.05 | 2.46 | 2.00 | 3.93 |
| Caudate Putamen | R | 8.70 | 3.75 | 4.10 | 0.00 | 14.74 |
| Extended Amygdala Central Division | R | 5.46 | 2.93 | 1.76 | -1.00 | 0.56 |
| Hypothalamus Lateral Zone | L | 5.03 | -1.41 | 1.76 | -1.00 | 0.54 |
| Reticular Thalamic Nucleus | R | 5.67 | 3.28 | 3.75 | -3.00 | 0.87 |
| Thalamus Ventral Posterior Complex | R | 4.90 | 2.58 | 3.28 | -4.00 | 0.78 |
| Zona incerta | R | 5.42 | 1.76 | 3.28 | -5.00 | 0.62 |
|  | L | 4.21 | -3.05 | 2.93 | -5.00 | 0.40 |
| Thalamus Posterior Nucleus | R | 4.68 | 2.11 | 4.45 | -6.00 | 0.78 |
| **Brainstem/Cerebellar Structures** | | |  |  |  |  |
| Corticospinal Tract | R | 6.15 | 3.75 | 3.28 | -4.00 | 0.71 |
| Trigeminothalmic Tract | L | 4.41 | -1.87 | 1.29 | -8.00 | 0.51 |
| Reticular Formation Midbrain | R | 4.27 | 2.23 | 4.45 | -8.00 | 0.44 |
| Inferior Colliculus | R | 4.72 | 0.12 | 6.44 | -9.00 | 0.84 |
|  | R | 4.37 | 0.23 | 6.80 | -10.00 | 0.62 |
| Raphe Nuclei Midbrain | L | 4.59 | -0.23 | 3.75 | -9.00 | 1.10 |
| Reticular Formation Pontomedullary | L | 7.38 | -1.41 | 0.47 | -10.00 | 1.21 |
| Trigeminal Nerve | R | 5.67 | 2.58 | 2.11 | -10.00 | 1.03 |
| Cerebellum Lobule 02 | R | 4.57 | 0.59 | 5.62 | -10.00 | 1.62 |
|  | R | 4.34 | 0.12 | 4.57 | -11.00 | 0.44 |
| Cranial Special Sensory Nuclei | R | 4.36 | 2.93 | 2.81 | -10.00 | 0.40 |
| Reticular Formation Medullary | L | 5.97 | -1.87 | 0.47 | -11.00 | 0.76 |
| Cerebellum Lobule 04 | R | 5.77 | 1.76 | 6.44 | -11.00 | 2.53 |

| **6. Autonomic Network** | | | | | | |
| --- | --- | --- | --- | --- | --- | --- |
| **Brain Region** | **Laterality** | **z-stat** | **X(mm)** | **Y(mm)** | **Z(mm)** | **Vol (mm^3^)** |
| **Cortical Structures** |  |  |  |  |  |  |
| Somatosensory Cortex Primary Jaw Region | R | 6.18 | 4.22 | 4.10 | 2.00 | 0.52 |
| Somatosensory Cortex Primary Barrel Field | R | 4.50 | 4.57 | 6.91 | -1.00 | 0.56 |
| Olfactory Cortex Lateral | R | 5.56 | 4.92 | 1.29 | -5.00 | 0.63 |
| Retrosplenial Cortex | R | 4.34 | 0.12 | 7.73 | -5.00 | 0.38 |
| **Subcortical Structures** |  |  |  |  |  |  |
| Caudate & Putamen | L | 5.32 | -2.34 | 5.27 | -1.00 | 0.88 |
|  | L | 4.59 | -2.69 | 6.09 | -1.00 | 0.43 |
| Thalamus Midline Nuclei | L | 4.10 | -0.59 | 4.57 | -2.00 | 0.44 |
|  | L | 4.63 | -0.59 | 2.46 | -3.00 | 2.28 |
| Thalamus Vental Lateral & Ventral Anterior Nuclei | R | 5.82 | 1.76 | 3.75 | -3.00 | 6.91 |
| Hypothalamus Lateral Zone | R | 6.66 | 0.47 | 2.11 | -4.00 | 4.02 |
|  | L | 5.23 | -0.23 | 1.29 | -4.00 | 3.78 |
| Thalamus Ventral Posterior Complex | R | 4.96 | 0.94 | 2.93 | -4.00 | 0.71 |
| Hippocampal Formation Dentate Gyrus | L | 4.72 | -2.23 | 6.09 | -4.00 | 0.88 |
| Hippocampal Formation CA1 Field | L | 4.60 | -3.05 | 7.27 | -4.00 | 0.60 |
|  | L | 4.12 | -2.23 | 7.62 | -4.00 | 0.52 |
|  | L | 6.52 | -1.05 | 6.09 | -5.00 | 3.80 |
|  | L | 4.97 | -1.76 | 6.44 | -5.00 | 0.48 |
| Thalamus Intralaminar Nuclei | R | 4.40 | 0.12 | 3.28 | -4.00 | 1.17 |
|  | L | 6.54 | -1.76 | 4.10 | -5.00 | 4.90 |
| Hippocampal Formation CA3 Field | L | 4.15 | -3.75 | 6.09 | -4.00 | 0.62 |
| Thalamus Posterior Nucleus | L | 4.83 | -2.69 | 5.27 | -4.00 | 1.81 |
| Pretectum | L | 5.94 | -1.05 | 5.27 | -5.00 | 1.28 |
| Cingulum | L | 4.23 | -2.34 | 8.09 | -5.00 | 0.43 |
| **Brainstem/Cerebellar Structures** |  |  |  |  |  |  |
| Superior Colliculus | L | 5.16 | -2.58 | 5.62 | -8.00 | 0.60 |
| Inferior Colliculus | L | 5.39 | -1.87 | 5.74 | -9.00 | 1.26 |
| Reticular Formation Medullary | L | 4.26 | -1.41 | 0.12 | -11.00 | 0.81 |

| **7. Cerebellar Network – Component 1** |  |  |  |  |  |  |
| --- | --- | --- | --- | --- | --- | --- |
| **Brain Region** | **Laterality** | **z-stat** | **X(mm)** | **Y(mm)** | **Z(mm)** | **Vol (mm^3^)** |
| **Cortical Structures** |  |  |  |  |  |  |
| Somatosensory Cortex Primary Dysgranular Region | L | 4.94 | -5.04 | 5.74 | 1.00 | 0.56 |
| Somatosensory Cortex Secondary | L | 4.46 | -6.56 | 4.45 | -1.00 | 0.59 |
| Somatosensory Cortex Primary Barrel Field | L | 4.66 | -5.86 | 5.62 | -1.00 | 0.67 |
| Retrosplenial Cortex | R | 5.04 | 0.12 | 8.44 | -7.00 | 2.05 |
|  | R | 4.51 | 0.12 | 7.73 | -8.00 | 1.81 |
| Corpus Callosum | R | 7.73 | 3.05 | 7.27 | -8.00 | 2.95 |
| Olfactory Cortex Lateral | R | 5.00 | 3.75 | 5.27 | -9.00 | 0.63 |
| **Subcortical Structures** |  |  |  |  |  |  |
| Caudate Putamen | R | 4.34 | 2.93 | 5.62 | 1.00 | 0.87 |
|  | L | 5.28 | -5.04 | 3.28 | -1.00 | 0.49 |
| Extended Amygdala Medial Division | R | 4.65 | 2.93 | 1.64 | -3.00 | 0.37 |
| Thalamus Lateral Nucleus | R | 4.14 | 2.93 | 4.80 | -4.00 | 0.49 |
| Hippocampal Formation Dentate Gyrus | L | 5.31 | -3.40 | 2.46 | -6.00 | 0.76 |
| Pretectum | R | 4.72 | 1.76 | 5.27 | -6.00 | 1.04 |
| **Brainstem/Cerebellar Structures** |  |  |  |  |  |  |
| Corticospinal Tract | L | 5.95 | -3.52 | 2.93 | -5.00 | 2.01 |
| Rubral Area | L | 5.52 | -1.41 | 3.28 | -5.00 | 1.50 |
| Inferior Colliculus | R | 5.97 | 0.59 | 7.62 | -10.00 | 2.68 |
| Simple Lobule | L | 4.24 | -4.22 | 6.09 | -10.00 | 0.38 |
|  |  |  |  |  |  |  |

| **8A. Thalamic-Brainstem Network – Component 1** | | | | |  |  |  |
| --- | --- | --- | --- | --- | --- | --- | --- |
| **Brain Region** | **Laterality** | | **z-stat** | **X(mm)** | **Y(mm)** | **Z(mm)** | **Vol (mm^3^)** |
| **Cortical Structures** | | |  |  |  |  |  |
| Olfactory Cortex Medial | R | | 5.17 | 0.12 | 3.28 | 2.00 | 3.45 |
| Motor Cortex Primary | R | | 3.43 | 1.76 | 8.09 | 2.00 | 1.80 |
| Motor Cortex Secondary | R | | 3.25 | 1.41 | 7.73 | 2.00 | 0.48 |
|  | R | | 3.99 | 0.94 | 8.09 | 1.00 | 0.80 |
| Somatosensory Cortex Secondary | L | | 4.46 | -6.33 | 3.63 | -2.00 | 3.24 |
|  | L | | 4.15 | -5.04 | 5.27 | -2.00 | 1.70 |
|  | L | | 3.44 | -5.86 | 4.80 | -2.00 | 2.03 |
| Corpus Callosum | R | | 3.86 | 0.94 | 7.27 | -4.00 | 0.66 |
| Parietal Cortex Posterior Area | L | | 4.81 | -3.05 | 8.55 | -5.00 | 1.98 |
| Visual Cortex Secondary | R | | 6.72 | 2.58 | 8.55 | -6.00 | 3.16 |
|  | R | | 5.98 | 3.28 | 8.55 | -6.00 | 0.92 |
| Visual Cortex Primary | R | | 3.24 | 3.40 | 8.09 | -8.00 | 0.62 |
| **Subcortical Structures** | | |  |  |  |  |  |
| Caudate Putamen | R | | 4.19 | 0.94 | 4.10 | 2.00 | 0.41 |
|  | R | | 3.49 | 1.29 | 4.10 | 1.00 | 0.54 |
|  | L | | 4.32 | -1.87 | 3.63 | 0.00 | 1.17 |
| Septal Region Lateral Group | L | | 7.02 | -0.70 | 3.28 | 1.00 | 2.05 |
| Hippocampal Formation CA2 Field | L | | 3.94 | -2.23 | 6.91 | -3.00 | 2.05 |
| Auditory Thalamus | L | | 3.68 | -2.69 | 4.92 | -6.00 | 1.25 |
| Hippocampal Formation Dentate Gyrus | L | | 3.12 | -3.40 | 2.11 | -6.00 | 0.37 |
| **Brainstem/Cerbellar Structures** | | | |  |  |  |  |
| Corticospinal Tract | L | | 3.87 | -4.22 | 5.62 | -3.00 | 1.32 |
| Rubral Area | R | | 7.01 | 0.12 | 3.63 | -6.00 | 42.23 |
| Reticular Formation Midbrain | L | | 2.92 | -1.87 | 4.10 | -7.00 | 0.40 |
|  | L | | 3.25 | -1.76 | 3.63 | -8.00 | 0.89 |
| Cerebellar Peduncle | L | | 2.88 | -3.05 | 1.76 | -8.00 | 0.48 |
|  | L | | 3.27 | -1.41 | 3.28 | -9.00 | 0.49 |
|  |  | |  |  |  |  |  |
| **8B. Thalamic-Brainstem Network – Component 2** | | | |  |  |  |  |
| **Brain Region** | **Laterality** | | **z-stat** | **X(mm)** | **Y(mm)** | **Z(mm)** | **Vol (mm3)** |
| **Cortical Structures** | | |  |  |  |  |  |
| Cingulate Cortex | R | | 3.27 | 0.94 | 5.27 | 3.00 | 0.54 |
|  | L | | 3.85 | -0.70 | 7.27 | 0.00 | 0.59 |
| Somatosensory Cortex Primary Jaw Region | L | | 4.21 | -5.04 | 6.09 | 2.00 | 0.58 |
| Motor Cortex Primary | L | | 4.06 | -1.87 | 8.55 | 2.00 | 0.52 |
| Somatosensory Cortex Primary Upper Lip Region | L | | 4.25 | -5.04 | 5.27 | 1.00 | 0.78 |
|  | R | | 3.70 | 5.04 | 5.27 | 1.00 | 1.04 |
| Cingulum | L | | 5.54 | -1.41 | 6.80 | 0.00 | 1.24 |
| Olfactory Tract Lateral | R | | 4.66 | 3.05 | 0.12 | -1.00 | 1.99 |
| Somatosensory Cortex Secondary | L | | 3.57 | -6.21 | 3.75 | -1.00 | 0.54 |
| Somatosensory Cortex Primary Barrel Field | L | | 3.41 | -5.04 | 5.27 | -1.00 | 1.33 |
| Insular Cortex | L | | 4.22 | -5.51 | 3.28 | -2.00 | 0.74 |
| Olfactory Cortex Lateral | L | | 5.38 | -4.22 | 0.59 | -3.00 | 3.43 |
|  | R | | 4.62 | 2.93 | 4.10 | -9.00 | 2.20 |
| Retrosplenial Cortex | L | | 3.22 | -0.23 | 8.09 | -3.00 | 0.56 |
|  | R | | 3.63 | 0.94 | 8.44 | -7.00 | 0.85 |
| Auditory Cortex Primary | L | | 3.04 | -6.68 | 5.27 | -7.00 | 1.44 |
| **Subcortical Structures** | | |  |  |  |  |  |
| Amygdaloid Nuclear Complex Laterobasal | R | | 4.48 | 3.40 | 0.59 | -2.00 | 1.83 |
| Thalamus Ventral Medial Nucleus | R | | 4.27 | 0.94 | 2.46 | -3.00 | 1.76 |
| Zona incerta | R | | 3.99 | 1.29 | 2.11 | -3.00 | 1.87 |
|  | L | | 3.59 | -2.23 | 2.93 | -5.00 | 0.85 |
| Extended Amygdala Central Division | R | | 3.72 | 3.75 | 2.46 | -3.00 | 1.07 |
|  | R | | 3.62 | 4.10 | 2.11 | -3.00 | 1.11 |
| Thalamus Ventral Posterior Complex | L | | 5.11 | -2.69 | 3.63 | -4.00 | 5.34 |
| Hippocampal Formatiom CA2 Field | L | | 3.15 | -4.22 | 5.62 | -5.00 | 0.87 |
| Hippocampal Formation Dentate Gyrus | R | | 6.27 | 3.40 | 5.27 | -7.00 | 3.03 |
| Hippocampal Formation Subicular Complex | L | | 3.27 | -3.87 | 6.91 | -7.00 | 0.54 |
|  | R | | 5.33 | 3.75 | 3.63 | -8.00 | 3.63 |
| Auditory Thalamus | L | | 3.23 | -3.87 | 4.45 | -7.00 | 0.49 |
| **Brainstem/Cerbellar Structures** | | | |  |  |  |  |
| Dorsal Column | L | | 4.25 | -1.52 | 2.93 | -6.00 | 0.81 |
| Rubral Area | R | | 3.63 | 0.94 | 4.10 | -6.00 | 0.70 |
| Transverse Pontine Fibres | L | | 8.42 | -1.41 | 0.47 | -7.00 | 53.39 |
| Reticular Formation Midbrain | L | | 3.44 | -1.41 | 4.10 | -8.00 | 1.88 |
| Vestibulocochlear Nerve | L | | 3.33 | -3.52 | 4.92 | -8.00 | 0.78 |
| Cerebellar Peduncle | L | | 3.22 | -3.87 | 2.46 | -9.00 | 0.70 |
| Cerebellum Lobule 02 | L | | 5.16 | -1.87 | 4.45 | -10.00 | 1.59 |
| Tegmental Nucleus Posterodorsal | L | | 4.02 | -0.59 | 3.28 | -10.00 | 0.88 |
| Nucleus Parabrachialis | L | | 3.06 | -3.05 | 3.75 | -10.00 | 0.63 |
|  |  | |  |  |  |  |  |
| **8C. Thalamic-Brainstem Network – Component 3** | | | | |  |  |  |
| **Brain Region** | **Laterality** | | **z-stat** | **X(mm)** | **Y(mm)** | **Z(mm)** | **Vol (mm^3^)** |
| **Cortical Structures** | | |  |  |  |  |  |
| Olfactory Cortex Medial | L | | 5.95 | -0.59 | 4.45 | 3.00 | 0.55 |
| Motor Cortex Primary | R | | 3.81 | 2.11 | 6.91 | 1.00 | 0.37 |
|  | R | | 4.36 | 1.76 | 8.55 | 0.00 | 1.76 |
| Somatosensory Cortex Primary Trunk Region | R | | 3.86 | 2.46 | 8.44 | -4.00 | 0.60 |
| Retrosplenial Cortex | R | | 3.96 | 2.93 | 6.91 | -9.00 | 0.96 |
| **Subcortical Structures** | | |  |  |  |  |  |
| Thalamus Anterior Nuclei | L | | 5.45 | -1.41 | 4.45 | -2.00 | 2.28 |
| Thalamus Mediodorsal Nucleus | L | | 3.80 | -1.05 | 4.10 | -3.00 | 0.62 |
| Stria Terminalis | L | | 4.68 | -4.69 | 4.10 | -4.00 | 0.87 |
| **Brainstem/Cerbellar Structures** | | | |  |  |  |  |
| Inferior Colliculus | R | 6.16 | | 0.94 | 5.74 | -9.00 | 4.44 |
| Basilar Pontine Nuclei | L | 6.12 | | -0.23 | 0.94 | -9.00 | 5.59 |
| Corticospinal Tract | L | 4.98 | | -0.35 | 0.12 | -9.00 | 1.04 |
| Olivary Complex Superior | R | 5.84 | | 0.94 | 0.47 | -10.00 | 2.24 |
| Reticular Formation Pontomedullary | L | 9.35 | | -1.05 | 0.47 | -10.00 | 18.03 |
| Reticular Formation Medullary | L | 5.30 | | -1.87 | 1.64 | -11.00 | 1.03 |
| Cerebellum Lobule 05 | R | 3.52 | | 1.41 | 7.27 | -11.00 | 0.44 |

**Table 1. Regional Brain Activity in Resting-State Networks of 2-Week Old Rat.** Table shows brain areas comprising 8 resting state networks in 2-week old rat (N=11; 5 female and 6 male from 4 different litters). Identified brain networks included **(1)** Default Mode, **(2)** Sensory (Exteroceptive), **(3)** Salience (Interoceptive), **(4)** Basal Ganglia-Thalamic-Hippocampal, **(5)** Basal Ganglia, **(6)** Autonomic, **(7)** Cerebellar, and **(8)** Thalamic-Brainstem networks. With the exception of the autonomic network and cerebellar networks, networks were comprised of multiple components. Maximal statistical value (z-stat), anatomical coordinates (x, y and z axes) and volumes (Vol) for each cluster of activity are provided for details of peak value, location and size (mm^3^), respectively. Z-axis values (mm) correspond to Bregma. Bregma of 0 mm corresponds to Panel 17 of the Rat Brain Atlas (Paxinos and Watson, 1998). Distribution of data for selected anatomical regions is listed separately for forebrain (cortical and subcortical) and brainstem/cerebellar structures for each component. *Abbreviations:* **L**, left; **R**, right.
